# Genome-Wide DNA Methylation in Early-Onset-Dementia Patients Brain Tissue and Lymphoblastoid Cell Lines

**DOI:** 10.3390/ijms25105445

**Published:** 2024-05-16

**Authors:** Oscar Ramos-Campoy, Aina Comas-Albertí, David Hervás, Sergi Borrego-Écija, Beatriz Bosch, Juan Sandoval, Laura Fort-Aznar, Fermín Moreno-Izco, Guadalupe Fernández-Villullas, Laura Molina-Porcel, Mircea Balasa, Albert Lladó, Raquel Sánchez-Valle, Anna Antonell

**Affiliations:** 1 Alzheimer’s Disease and Other Cognitive Disorders Unit, Neurology Service, Hospital Clínic de Barcelona, FRCB-IDIBAPS, Universitat de Barcelona (UB), 08036 Barcelona, Spainacomasa@recerca.clinic.cat (A.C.-A.); borrego@clinic.cat (S.B.-É.); bbosch@recerca.clinic.cat (B.B.); fort@recerca.clinic.cat (L.F.-A.); gfernanv@clinic.cat (G.F.-V.); lmolinap@clinic.cat (L.M.-P.); mbalasa@clinic.cat (M.B.); allado@clinic.cat (A.L.); 2 Department of Applied Statistics and Operations Research and Quality, Universitat Politècnica de València, 46022 Valencia, Spain; daherma@eio.upv.es; 3 Epigenomics Core Facility, Health Research Institute La Fe, 46026 Valencia, Spain; epigenomica@iislafe.es; 4 Cognitive Disorders Unit, Department of Neurology, Hospital Universitario Donostia, 20014 San Sebastian, Spain; fermin.morenoizco@osakidetza.eus; 5 Instituto de Investigación Sanitaria Biogipuzkoa, Neurosciences Area, Group of Neurodegenerative Diseases, 20014 San Sebastian, Spain; 6 Neurological Tissue Bank, Biobank-Hospital Clinic-IDIBAPS, 08036 Barcelona, Spain; 7 Facultat de Medicina i Ciències de la Salut, Institut de Neurociències, Universitat de Barcelona (UB), 08036 Barcelona, Spain

**Keywords:** Alzheimer’s disease, frontotemporal dementia, lymphoblastoid cell lines, brain tissue, DNA methylation, diagnostic signature, epigenetic assessment

## Abstract

Epigenetics, a potential underlying pathogenic mechanism of neurodegenerative diseases, has been in the scope of several studies performed so far. However, there is a gap in regard to analyzing different forms of early-onset dementia and the use of Lymphoblastoid cell lines (LCLs). We performed a genome-wide DNA methylation analysis on sixty-four samples (from the prefrontal cortex and LCLs) including those taken from patients with early-onset forms of Alzheimer’s disease (AD) and frontotemporal dementia (FTD) and healthy controls. A beta regression model and adjusted *p*-values were used to obtain differentially methylated positions (DMPs) via pairwise comparisons. A correlation analysis of DMP levels with Clariom D array gene expression data from the same cohort was also performed. The results showed hypermethylation as the most frequent finding in both tissues studied in the patient groups. Biological significance analysis revealed common pathways altered in AD and FTD patients, affecting neuron development, metabolism, signal transduction, and immune system pathways. These alterations were also found in LCL samples, suggesting the epigenetic changes might not be limited to the central nervous system. In the brain, CpG methylation presented an inverse correlation with gene expression, while in LCLs, we observed mainly a positive correlation. This study enhances our understanding of the biological pathways that are associated with neurodegeneration, describes differential methylation patterns, and suggests LCLs are a potential cell model for studying neurodegenerative diseases in earlier clinical phases than brain tissue.

## 1. Introduction

Currently, more than 55 million people have dementia worldwide, and every year, there are nearly 10 million new cases. Alzheimer’s disease (AD), the most frequent type of neurodegenerative dementia [1], can be classified into early-onset and late-onset forms. In early-onset AD (EOAD) (5% of AD cases), symptoms appear, according to consensus, before 65 years old [2]. According to the etiology, the vast majority of EOAD cases have a sporadic origin (sEOAD) [3], but there is also a small percentage of cases (<1%) that show an autosomal dominant pattern of inheritance (ADAD), which is caused by mutations in three genes involved in the β-amyloid cascade: *APP*, *PSEN1*, and *PSEN2* [4].

Frontotemporal dementia (FTD) refers to a group of neurodegenerative disorders comprising different clinical syndromes ranging from behavioral to motor forms [5]. The neuropathology of FTD is also heterogeneous, with diverse protein aggregates, among which the most frequent are tau, TDP43, and FET-related proteins. One-third of patients have an autosomal dominant pattern of inheritance (gFTD), with *C9orf72*, *GRN*, and *MAPT* being the most frequent genes involved [6].

Epigenetic mechanisms, potential underlying pathogenic mechanisms of neurodegenerative diseases, have been within the scope of several studies performed so far. The most-studied epigenetic modification is DNA methylation at cytosines in high-density cytosine–guanine sequences (CpGs), referred to as CpG islands, carried out by DNA methyltransferases [7]. Most studies investigating DNA methylation status in AD have been performed on brain tissue and late-onset forms of AD, reporting differences in methylation, mainly hypermethylation concerning controls, dysregulating the expression of genes involved in myelination, synaptic transmission, or immune response [8,9,10,11]. Nonetheless, there is growing evidence that methylation is also deregulated in peripheral blood in patients with neurodegenerative diseases, and this possibility is attracting increasing attention [12,13]. In regard to FTD, several studies have explored the effects of methylation changes on brain or blood in some of the gFTD-causative genes, describing that promoter hypermethylation in *GRN* and *C9orf72* genes leads to a decrease in gene expression [14,15,16,17,18,19]. A few works have compared more than one type of dementia to evaluate similarities and differences regarding physiopathological mechanisms related to neurodegenerative diseases [20,21,22,23].

Although the brain is the target organ for studying neurodegenerative diseases, lymphoblastoid cell lines (LCLs; B lymphocytes immortalized with Epstein–Barr virus) have been used as an alternative model to brain tissue to study several aspects of neurodegenerative diseases [24,25,26,27]. They are much easier to obtain, can be acquired at earlier stages of disease, and represent a cell culture model. To the best of our knowledge, no study has examined and compared global gene methylation in diverse neurodegenerative diseases in LCLs until now. However, some publications have analyzed methylome changes in peripheral blood mononuclear cells (PBMCs), finding greater global DNA methylation in AD patients compared to healthy controls [28]. Moreover, regarding FTD, several studies using LCLs have focused on surveying epigenetic changes in genes causative of this disease’s genetic forms, mainly *C9orf72* and *GRN*, revealing an inverse correlation between their promoter methylation levels and gene expression [15,29].

This study aimed to analyze and compare the genome-wide methylation profiles of sporadic and genetic forms of early-onset AD and FTD patients in two types of samples: frozen prefrontal cortex tissue and LCLs. Moreover, using the results obtained from our previous study [30], we performed a correlation analysis between methylation and gene expression data from the same cohort.

## 2. Results

The principal component analysis (PCA) showed clear separation between the brain and LCL samples. Therefore, separate analyses were conducted for each sample. No outliers were detected after data normalization and PCA analysis.

**Figure 1:**
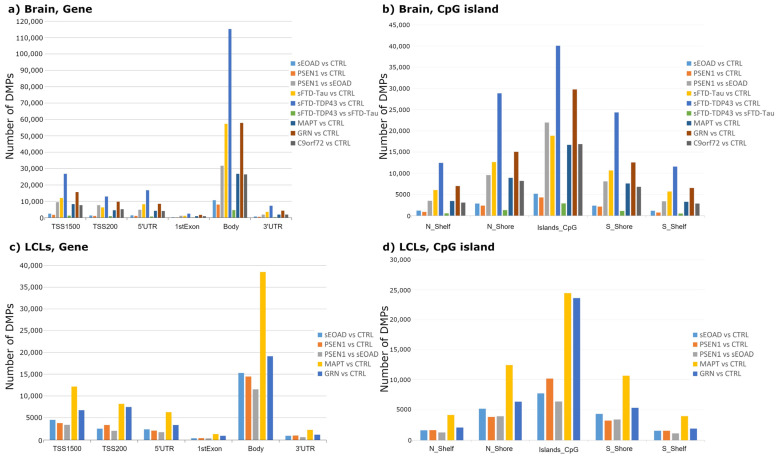
Distribution of the differentially methylated positions (DMPs) found in brain (**a**,**b**) and LCL (**c**,**d**) comparisons. Bar plot depicting the number of brain DMPs found in each region related to gene (**a**,**c**) and CpG islands (**b**,**d**). CpGs sites were found in different gene regions: transcription start site (TSS1500 and TSS200), untranslated region (5′UTR and 3′UTR), first exon, and body gene. If we take CpGs islands as reference, CpGs may be found in shores (2 kb from islands) or shelves (5 kb from islands) and may be closer to 5′ end (N) or 3′ end (S). Filters applied: adjusted-*p* value < 0.05. Abbreviations: CTRL, healthy controls; sEOAD, sporadic early-onset Alzheimer’s disease; PSEN1, autosomal dominant Alzheimer’s disease caused by mutation in *PSEN1* gene; MAPT, GRN, and C9orf72, familial frontotemporal dementia caused by mutation in *MAPT*, *GRN*, or *C9orf72* genes; sFTD-Tau, sporadic frontotemporal dementia with tau deposits; sFTD-TDP43, sporadic frontotemporal dementia with TDP43 deposits; LCLs, lymphoblastoid cell lines.

All comparisons revealed several DMPs mainly located in CpG islands, progressively decreasing in adjacent regions. Based on the relative position to the gene location, the gene body was the area with more DMPs, followed by the transcription start site 1500 (TSS1500) (Figure 1). The total number of DMPs are included in Supplemental Material S3. Overall, our statistical analyses found more DMPs in LCL comparisons of patients compared to controls than in the same comparisons in brain tissue, except for the *GRN* vs. CTRL comparison.

We also looked for epigenetic changes in genes causative of dementia, comparing mutation carriers for the gene with controls (Supplemental Material S4). There was only one CpG with an absolute Beta difference value > 0.2; it was located in the promoter region of the *MAPT* gene (cg24801230) and hypermethylated in patients from the MAPT group.

### 2.1. Alzheimer’s Disease

**Figure 2:**
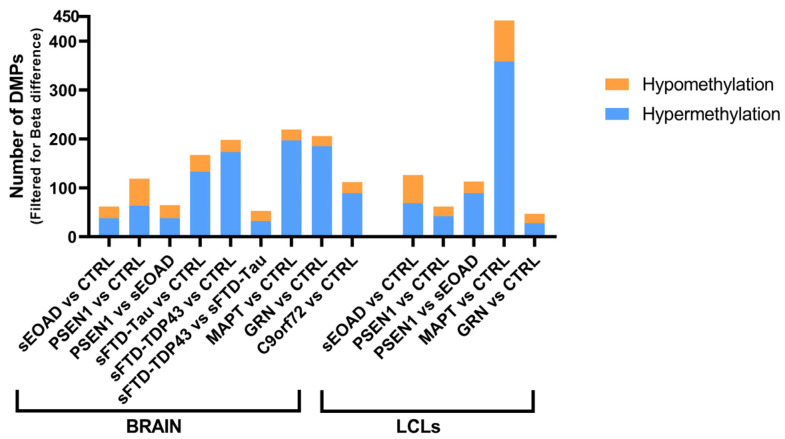
Number of differentially methylated CpGs (DMPs). Bar plot showing the quantities of hyper- and hypomethylated CpGs found in each comparison performed per tissue. Filters applied: in the brain, adjusted-*p* value < 0.05 and absolute value of Beta difference > 0.25; in LCLs, adjusted-*p* value < 0.01 and absolute value of Beta difference > 0.35. Abbreviations: CTRL, healthy controls; sEOAD, sporadic early-onset Alzheimer’s disease; PSEN1, autosomal dominant Alzheimer’s disease caused by mutation in *PSEN1* gene; MAPT, GRN, and C9orf72, familial frontotemporal dementia caused by mutation in *MAPT, GRN*, or *C9orf72* genes; sFTD-Tau, sporadic frontotemporal dementia with tau deposits; sFTD-TDP43, sporadic frontotemporal dementia with TDP43 deposits; LCLs, lymphoblastoid cell lines.

The Beta regression model after filtering for adjusted-*p* value and Beta difference retrieved the DMPs shown in Figure 2. In all AD-patients-versus-healthy controls comparisons performed on both tissues, there were more hypermethylated DMPs than hypomethylated DMPs.

**Figure 3:**
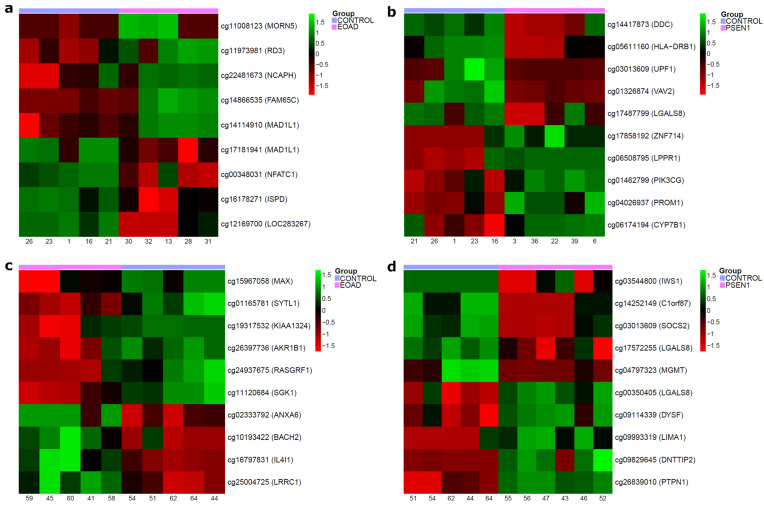
Heatmaps with the top 10 differentially methylated positions (DMPs) in each AD comparison performed. For each CpG, the associated gene is given in parentheses. (**a**) Brain sEOAD vs. CTRL; (**b**) brain PSEN1 vs. CTRL; (**c**) LCL sEOAD vs. CTRL; (**d**) LCL PSEN1 vs. CTRL. Abbreviations: CTRL, healthy controls; EOAD, sporadic early-onset Alzheimer’s disease; PSEN1, autosomal dominant Alzheimer’s disease caused by mutation in *PSEN1* gene; LCLs, lymphoblastoid cell lines.

We then selected the 10 DMPs with the highest Beta difference values for each comparison regarding the brain and LCL samples (Figure 3 and Supplemental Material S5).

#### 2.1.1. Common DMPs in AD Subtypes

**Figure 4:**
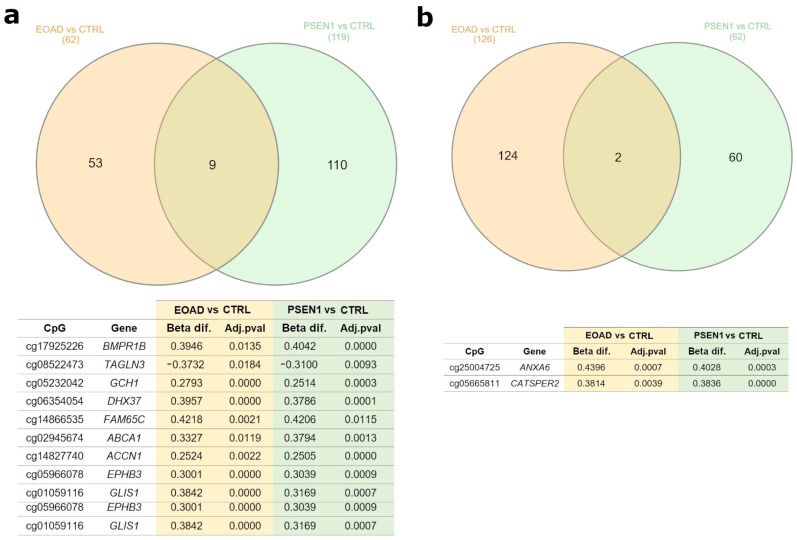
Common differentially methylated CpGs (DMPs) found in different AD comparisons. Venn diagrams showing common DMPs in brain (**a**) and LCL comparisons (**b**). Underneath each Venn diagram, a table shows the common DMPs found and their corresponding gene, Beta difference (Beta dif.), and adjusted-*p* value (Adj.pval). Filters applied: in brain, adjusted-*p* value < 0.05 and absolute value of Beta difference > 0.25; in LCLs, adjusted-*p* value < 0.01 and absolute value of Beta difference > 0.35. Abbreviations: CTRL, healthy controls; sEOAD, sporadic early-onset Alzheimer’s disease; PSEN1, autosomal dominant Alzheimer’s disease caused by mutation in *PSEN1* gene; LCLs, lymphoblastoid cell lines.

We found nine common DMPs in the brain in the AD patients (genetic and sporadic) versus control comparisons. The DMPs were found in the genes *BMPR1B*, *TAGLN3*, *GCH1*, *DHX37*, *FAM65C*, *ABCA1*, *ACCN1*, *EPHB3*, and *GLIS1*. In the patients, all of these genes were hypermethylated except for *TAGLN3*, which was hypomethylated. On the other hand, in LCLs, there were only two DMPs in common in the same group comparisons (located in the genes *ANXA6* and *CATSPER2*), both of which were hypermethylated in the patients (Figure 4).

#### 2.1.2. Biological Significance Analysis

Analysis of biological significance found several significant pathways (from Reactome) and biological processes (BPs; from GO-BP) altered in all AD comparisons. Neural system pathways were found in brain tissue, including with respect to BPs such as axon development (PSEN1 vs. CTRL). In LCLs, neurotransmitter receptors and long-term potentiation were obtained in sEOAD vs. CTRL comparisons by applying Reactome database.

Metabolic disturbances were also a common outcome in all the comparisons performed, affecting carbohydrate metabolism (all brain comparisons) or phosphorylation regulation (all LCL comparisons). Immune system pathways were present, in both tissues, affecting cytokine signaling and adaptive response. Finally, pathways related to the *MECP2* gene expression node and its regulation of neural cell terms were found in both groups of patients in LCLs. The complete list of BPs and pathways can be found in Supplemental Materials S6 and S7.

### 2.2. Frontotemporal Dementia

The Beta regression model results were filtered by adjusted-*p* value and Beta difference variables and a shorter list of DMPs was obtained, shown in Figure 2. Hypermethylation was the most frequent alteration in the brain samples from the patients, in comparison withhealthy controls.

**Figure 5:**
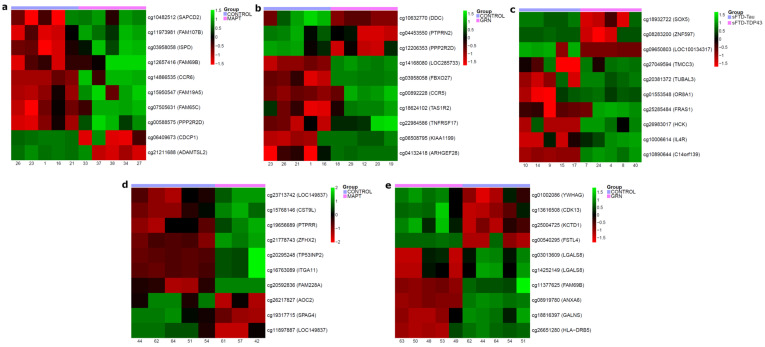
Heatmaps with the top 10 differentially methylated positions (DMPs) in some of the FTD comparisons performed. Each CpG obtained has its associated gene beside it. (**a**) Brain MAPT vs. CTRL; (**b**) brain GRN vs. CTRL; (**c**) brain sFTD-TDP43 vs. sFTD-Tau; (**d**) LCLs MAPT vs. CTRL; (**e**) LCLs GRN vs. CTRL. Abbreviations: CTRL, healthy controls; MAPT and GRN, familial frontotemporal dementia caused by mutation in *MAPT* or *GRN* genes; sFTD-Tau, sporadic frontotemporal dementia with tau deposits; sFTD-TDP43, sporadic frontotemporal dementia with TDP43 deposits; LCLs, lymphoblastoid cell lines.

We also selected the 10 DMPs with the highest Beta difference for each comparison in the brain and LCLs samples (Figure 5 and Supplemental Materials S5 and S8).

#### 2.2.1. Common DMPs in FTD Subtypes

**Figure 6:**
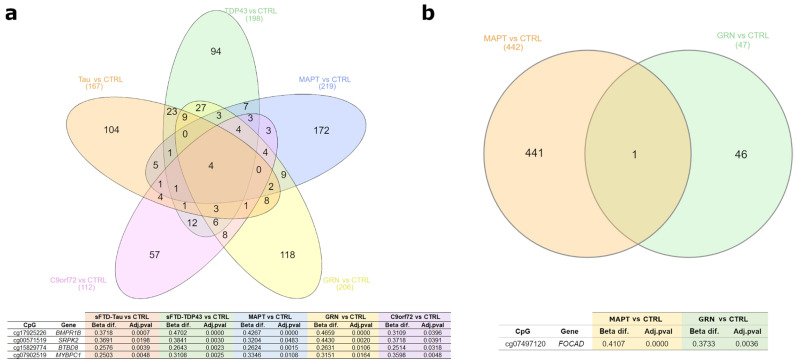
Common differentially methylated CpGs (DMPs) found between different comparisons in FTD. Venn diagrams showing common DMPs in brain (**a**) and LCL comparisons (**b**). The table underneath the Venn diagram (**a**) shows the common DMPs found and their correspondent genes, Beta differences (Beta dif.), and adjusted-*p* values (Adj.pval). Filters applied: in brain, adjusted-*p* value is < 0.05 and absolute value of Beta difference is >0.25; in LCLs, adjusted-*p* value is <0.01 and absolute value of Beta difference is >0.35. Abbreviations: CTRL, healthy controls; MAPT, GRN, and C9orf72, familial frontotemporal dementia caused by mutation in *MAPT*, *GRN*, or *C9orf72* genes; sFTD-Tau, sporadic frontotemporal dementia with tau deposits; sFTD-TDP43, sporadic frontotemporal dementia with TDP43 deposits; LCLs, lymphoblastoid cell lines.

We found four common DMPs between all the FTD-patient-subtype-versus-healthy controls comparisons involving brain tissue. These DMPs were located in the genes *BMPR1B*, *SRPK2*, *BTBD8*, and *MYBPC1*, all of which were hypermethylated in the patients (Figure 6). By comparing the three genetic patient groups with the controls, we found 12 shared DMPs. On the other hand, comparing the two sporadic patient groups with the controls showed 42 DMPs in common. These DMPs were mostly hypermethylated in the patients (39/42), and there was a complete concordance about the hyper- or hypomethylation status both in sFTD with tau deposits and sFTD with TDP43 deposits (Supplemental Material S9).

#### 2.2.2. Biological Significance Analysis

Biological significance analysis of the FTD groups comparisons also identified significant GO-BPs and pathways from the Reactome resource, except for the brain C9orf72 vs. CTRL comparison, which did not show any significant GO-BPs. Nervous system BPs were frequently found in all sporadic and genetic patients versus healthy controls comparisons regarding brain tissue, as well as in the LCL comparison of the MAPT vs. CTRL groups. They were specifically related to neuron development and differentiation.

Deregulated immune system pathways were found in the sporadic and genetic FTD groups with TDP43 deposition in both tissues. Signal transduction and protein metabolism pathways were found in the sporadic and genetic groups with tau deposition. The complete lists of the BPs and pathways found are included in Supplemental Materials S6 and S7.

### 2.3. Common Differentially Methylated CpGs in AD and FTD

Just one common DMP was found in all the patients-versus-controls comparisons for brain tissue, namely, cg17925226, which is located in the promoter region of the *BMPR1B* gene and was hypermethylated in all patientsgroups. No common DMPs were obtained in any of the patients-versus-control comparisons regarding LCLs. The list of common DMPs obtained can be found in Supplemental Material S10.

### 2.4. Diagnostic Signatures

We applied an Elastic Net model to obtain a list of CpGs that allowed differentiation of the groups for each pairwise comparison of patients versus controls. These lists of CpGs have a diagnostic signature potential when analyzed all together in the groups of interest (Supplemental Material S11).

### 2.5. Pyrosequencing Validation

The DMPs selected for validation were located in the *TUBAL3* and *ABCA1* genes. After performing non-parametric statistical analysis (using the Mann–Whitney U test for *TUBAL3* and the Kruskal–Wallis test for *ABCA1*), both of them were validated, as shown in Supplemental Material S2. The DMP in *TUBAL3* was significant when comparing both groups of sporadic FTD, while the DMP in *ABCA1* was significant in the sporadic and genetic AD brain groups compared to healthy controls.

### 2.6. Correlation Analysis

A correlation analysis between gene expression and DNA methylation microarrays data from the same individuals was performed, considering the DMPs for each comparison. Significant results (adjusted-*p* value < 0.05, absolute value of correlation coefficient > 0.7 and absolute value of Beta difference > 0.1) are shown in Table 1.

In brain tissue, all DMPs exhibited a negative correlation between DNA methylation levels and gene expression. We found two CpGs in the GRN vs. CTRL comparison (cg24203376 within the *TDRD1* gene and cg05726248 within the *TESPA1* gene) and one CpG common to both comparisons of sporadic FTD patients versus healthy controls (cg12150421 within the *KIF17* gene).

Unlike in brain tissue, in LCLs, the majority of DMPs had a positive correlation between DNA methylation and gene expression. We detected two CpGs (cg17369694 and cg01341801) correlating with *HLA-DRB5* expression in three comparisons: sEOAD vs. CTRL, MAPT vs. CTRL, and GRN vs. CTRL. Moreover, two CpGs (cg22730830 and cg01232511) correlated with *PRSS21* gene expression, found in sEOAD vs. CTRL and MAPT vs. CTRL, which had a negative correlation. Other significant results are presented in Table 1.

## 3. Discussion

Here, we present the results of a genome-wide DNA methylation study conducted on groups of patients with familial and sporadic forms of early-onset AD and FTD using two types of samples: brain prefrontal cortex tissue and LCLs.

Previous evidence regarding methylation alterations in brain samples from AD patients has exhibited some degree of variability. Several studies, mainly centered on late-onset AD, reported an increased level of methylation in the frontal cortex [31,32,33], an observation that is consistent with our findings. This hypermethylation does not seem region-dependent since other brain areas, like the hippocampus or entorhinal cortex, also exhibited hypermethylation [31,34]. However, there are also reports indicating that hypomethylation was more commonly observed [9,35]. The variability in the results could be attributed to the varying cellular compositions of the studied samples, as there is evidence suggesting differences in the degree of methylation among different types of nervous system cells [36]. Another potential contributing factor is the dynamic nature of methylation, as epigenetic mechanisms are not static and may undergo changes influenced by environmental factors or aging [37].

Few researchers have used lymphoblastoid cell lines (LCLs) from AD patients to investigate DNA methylation changes. Instead, several studies have employed peripheral blood mononuclear cells (PBMCs) or whole blood, with some of them comparing DNA methylation patterns between brain tissue and whole blood [13,38,39,40].

Our findings obtained from FTD patients also revealed hypermethylation to be the most common observation in brain samples. To the best of our knowledge, there are no previous reports analyzing the whole-genome methylation statuses of FTD patients, either in brain tissue or in LCLs. Previous studies using brain samples have specifically investigated the methylation statuses of genes implicated in FTD, particularly *C9orf72* and *GRN*. Other studies have employed whole-blood samples obtained from sporadic FTD patients, but a consensus regarding the predominance of hyper- or hypomethylation compared to controls has not been reached. Some studies have reported increased methylation in FTD patients compared to controls [41], while others have found hypomethylation to be more prevalent in patients [42].

### 3.1. Alzheimer’s Disease

We searched for the top DMPs with the highest Beta difference values, obtained in each of the comparisons of patients versus controls. In brain tissue, among these CpGs, it is important to highlight that some of them are located in genes involved in immune response function. One of these DMPs, found in the brain PSEN1 vs. CTRL comparison, is a CpG related to the *HLA-DRB1* gene. It is a member of the major histocompatibility complex and plays an important role in immune response regulation. Prior studies have defined it as a susceptibility gene for developing AD, particularly in early-onset forms [43,44]. The top methylated DMPs in LCLs also showed interesting results, since many DMPs were related to genes that are somehow associated with AD. In the sEOAD vs. CTRL comparison, we found the *SGK1* gene, which seems to act as a survival factor and whose expression has been reported to be increased in AD patients [45]. A DMP associated with *DYSF*, another gene reported to be overexpressed in AD patients [46], was found in the PSEN1 vs. CTRL comparison.

Regarding common DMPs in sporadic and genetic patients, we found nine DMPs in common in brain tissue and two DMPs in common in LCLs. Some of the common DMPs found in the brain are within genes known to confer a risk for developing AD, like *GLIS1*, a regulator of transcription, or *ABCA1*, a membrane transporter [47,48]. Interestingly, another DMP was related to the *GCH1* gene, which encodes a key enzyme in dopamine synthesis and whose variants have been reported to be a risk factor for Parkinson’s disease [49]. In LCLs, one of the two DMPs in common is located in the *ANXA6* gene, which belongs to a family of genes whose proteins regulate the interface between the membrane and cytoplasm. *ANXA6* has been shown to interact with axonal tau protein, contributing to its pathological distribution [50]. We also searched for common DMPs between different tissues and in the same comparison, but we obtained few matches.

The most frequently altered biological pathways in brain tissue were related to the metabolism of carbohydrates, steroids, or catabolic process regulation. Metabolic disturbances are a known feature in AD, recognized not only in gene expression studies [51] but also in those that analyze methylome differences [52]. The fact that metabolism dysregulation was found both in sporadic and genetic patients makes it a common element in the evolution of neurodegeneration and establishes metabolism as a key factor for cellular survival. In our study, other altered pathways found in genetic patients were those associated with neural development and neurotransmitter regulation. Nervous system development or synaptic transmission pathways have been found in previous studies [9,52,53], and there is evidence of some variable methylated regions being over-represented in these pathways, which have been reported to correlate with AD neuropathology [54].

In LCLs, pathways related to metabolic processes were observed, and they were similar to those found in the brain. However, there is limited evidence regarding the biological processes associated with epigenetic changes in LCLs. The presence of these changes in patients at an early stage of this disease may suggest that they have been altered since the onset of the neurodegenerative process. Pathways related to *MECP2* gene function were identified in both sporadic and genetic LCLs AD patients’ comparisons. This gene encodes for a protein capable of binding methylated DNA and acting as a transcriptional repressor [55]. Although it is primarily linked to autism spectrum disorders, studies on animal models have indicated a potential association between *MECP2* alterations and AD pathogenesis, as well as cognitive decline [56,57,58,59]. Moreover, recent research on humans suggests that *MECP2* may unveil a novel etiopathogenetic mechanism of sporadic AD [60].

### 3.2. Frontotemporal Dementia

We examined the top DMPs in the comparisons of sporadic FTD patients, which were ranked by Beta difference values. None of the top ten DMPs identified in the sporadic FTD group overlapped with the top 10 DMPs found in the AD groups. However, we identified numerous genes previously implicated in other neurodegenerative diseases, although not specifically in FTD. This suggests that there are potential shared molecular mechanisms among these diseases. One of these genes was *CUL3*, found in the sFTD-Tau vs. CTRLs comparison, a gene with multiple functions in cell cycle regulation, synaptic control, and proteasomal degradation that has recently been found to be downregulated in AD animal models [61,62]. The *DNAJB6* gene showed a significant difference between individuals with sFTD-TDP43 and CTRLs. This gene has been reported to be dysregulated in Parkinson’s disease and multiple system atrophy [63], and there is a reported case of FTD caused by its mutation [64]. Upon comparing both types of sporadic FTD patients, we found a DMP in the *SOX5* gene, a gene involved in corticospinal motor neuron development and a known risk factor for developing amyotrophic lateral sclerosis (ALS) [65]. As ALS is part of the ALS-FTD clinical spectrum and shares the most common cause of disease with FTD (G4C2-repeat expansion in *C9orf72*), this finding could be attributed to common underlying mechanisms. Additionally, anotherDMP within the *TUBAL3* gene was identified when comparing both types of sporadic FTD patients, and this finding was validated using pyrosequencing.

Regarding the genetic FTD group comparisons, some of the top ten DMPs were shared with the top DMPs identified in the sporadic and familial AD patients compared to the controls. An example is the differentially methylated position (DMP) associated with the *HLA-DRB1* gene, which was identified in the *C9orf72* group. Previous reports have indicated that this gene is downregulated in FTD [66]. Our data indicate the hypermethylation of a DMP located within the gene body, which likely results in decreased expression. Similarly, *PTPRN2*, which encodes a transmembrane protein involved in neurotransmitter secretion, was found to be hypermethylated in the *GRN* group. There is evidence suggesting decreased expression of *PTPRN2* in both AD and FTD [67]. Lastly, in the *C9orf72* group, we observed altered methylation of the *TP73* gene, variants of which have been linked to ALS and FTD [68,69].

In LCLs from genetic FTD patients, we also identified the top DMPs in common with AD, which were associated with the *LGAL8*, *ANXA6*, or *HLA-DRB1* genes. Other DMPs found were within the genes *HLA-DRB5* or *YWHAG*, which have also been identified as risk factors in the development of FTD and Parkinson’s disease, respectively [70,71].

To the best of our knowledge, only a few reports have studied altered pathways in FTD using genome-wide methylome analysis. In this cohort, we observed that neuronal development and differentiation pathways were commonly disrupted in FTD brains and were present in the majority of the patient-versus-control comparisons, a result that is consistent with findings from other studies [41]. Immune pathways were dysregulated in the *GRN* and sFTD-TDP43 groups compared to the controls, particularly those related to cytokine release and the adaptive immune system. These results suggest a potentially significant role of inflammation in FTD characterized by TDP43 deposition. Nonetheless, in patients with tau deposition, we found metabolic dysregulation and numerous pathways related to altered signaling and *ERBB* family genes, which encode for tyrosine kinase receptors and have been identified as risk factors for developing ALS or FTD [72].

In LCLs, we were able to analyze the MAPT and GRN groups, and we found altered neural pathways and cytokine signaling pathways, both of which were also found in the FTD brain tissue results. Finally, like in the LCLs in AD comparisons, we found many dysregulated pathways related to the *MECP2* gene, which may point towards altered neuron maturation and toxicity in FTD as well [56].

### 3.3. Correlation Analysis

The correlation analysis between the gene expression array data and methylome array data from the same cohort showed interesting correlations between differential CpGs methylation status and gene expression. We found a negative correlation between the methylation grade of cg12150421 and the expression of the *KIF17* gene in brain tissue, a correlation observed in both groups of sporadic FTD patients. *KIF17* is responsible for transporting cargo along microtubules. In LCLs, we found more CpGs that exhibited a significant correlation with expression levels than in brain tissue. Some of these CpGs were shared between AD and FTD patients (sEOAD, MAPT, and GRN groups versus healthye controls). For instance, we identified two DMPs (cg17369694 and cg01341801) associated with the same gene, *HLA-DRB5*. Throughout all the mentioned comparisons, we observed that both CpGs were hypomethylated and displayed a positive correlation, suggesting that *HLA-DRB5* expression levels are lower in LCLs of these groups of patients.

One limitation of our study is the relatively limited number of subjects in each subgroup, which could have constrained the statistical power of the analysis. Nonetheless, we have obtained results with significant adjusted-*p* values. Another limitation is the unavailability of LCLs from sporadic FTD and *C9orf72* groups, which precludes comparisons in both tissues. Regarding the use of LCLs, despite the immortalized nature of this cell culture model, the main aim of this study was to compare data obtained from this biological sample with brain tissue data and evaluate the former as a plausible biological model for the study of neurodegenerative diseases. Lastly, our study focused on DNA methylation, an epigenetic mechanism that can modulate gene expression at a pre-transcriptional level. However, other epigenetic modifications, such as chromatin modifications and changes in regulatory RNAs (miRNA), are also relevant for the development of AD. For instance, microRNAs (miRNAs) regulate gene expression at the post-transcriptional level, thereby repressing mRNA translation, and they have been extensively studied in relation to AD [73]. Moreover, histone modifications such as lysine acetylation or arginine/lysine methylation represent post-translational epigenetic modifications that activate or repress gene transcription [74,75]. So, considering these factors collectively is essential for obtaining a comprehensive understanding of chromatin compaction and how it regulates gene transcription.

In conclusion, our study presents data on genome-wide DNA methylation in a broad spectrum of AD and FTD subtypes, revealing potential targets for future biomarkers or therapeutic strategies and identifying altered biological pathways. Additionally, we provide evidence supporting the usefulness of LCLs in neurodegenerative diseases research. As future research steps, we would like to determine if differential methylation is translated to differential gene expression for some of the most interesting genes regarding neurodegenerative diseases and to validate some differential methylation findings obtained from whole blood, specifically those found in LCLs.

## 4. Materials and Methods

### 4.1. Samples and Clinical Data

We selected 64 samples from two different tissues: frozen prefrontal cortex (*n* = 40) and LCLs (*n* = 24). Frozen prefrontal cortex tissue was obtained from the Neurological Tissue Bank of IDIBAPS-Hospital Clínic de Barcelona (NTB-IHC) (*n* = 38) and Basque Biobank-Biodonostia (*n* = 2). We included brain samples obtained from healthy controls (CTRL; *n* = 5), sEOAD (*n* = 5) and patients with ADAD due to mutations in *PSEN1* gene (PSEN1; *n* = 5), genetic FTD caused by mutations in *MAPT*, *GRN* or *C9orf72* genes (MAPT *n* = 5; GRN *n* = 5; C9orf72 *n* = 5), and sporadic FTD with tau deposits (sFTD-Tau; *n* = 5) and TDP43 deposits (sFTD-TDP43; *n* = 5). All brain donors had died in advanced clinical stages after several years of disease duration.

LCLs were obtained from subjects who visited the Alzheimer’s disease and other cognitive disorders Units of Hospital Clínic de Barcelona, Barcelona, Spain (*n* = 24). The quantities of samples included from each group are as follow: CTRL *n* = 5; sEOAD *n* = 5; PSEN1 *n* = 6; MAPT *n* = 3; and GRN *n* = 5. *C9orf72* mutated samples and sporadic FTD samples were not available. All AD patients (both sEOAD and PSEN1 groups) had their diseases biologically confirmed using cerebrospinal fluid biomarkers and presented a typical amnestic phenotype. To stage dementia severity, we applied the Clinical Dementia Rating scale—Global Score (CDR-GS). In sEOAD group, 2 out of 5 patients had a CDR-GS of 0.5, while the other 3 had a CDR-GS of 1. In the PSEN1 group, the majority of subjects had a CDR-GS of 1 (4/6); one had a CDR-GS of 0.5, and the last one had a CDR-GS of 2. Genetic FTD patients developed behavioral variant FTD. Only one *MAPT* mutation carrier was diagnosed with semantic dementia, which eventually led to behavioral variant FTD. Regarding dementia staging, in the MAPT group, 2 out of 3 patients had a CDR-GS of 0.5, and the rest had a CDR-GS of 3. Finally, in the GRN group, 3 out of 5 subjects had a CDR-GS of 2, and the remaining two had a CDR-GS of 3.

Demographics and other relevant variables are shown in Table 2. Considering all pairwise comparisons performed for each tissue, no significant differences in any variable were found. More information about patients harboring genetic mutations is shown in Supplemental Material S1.

This study was performed in line with the principles of the Declaration of Helsinki. All patients had signed informed consent forms for sample donation. This project was approved by the local Ethics Committee of the Hospital Clínic de Barcelona.

### 4.2. Lymphoblastoid Cell Line Isolation and Culturing

Cell lines were grown in suspension in upright T-25 flasks (Sarstedt, Nümbrecht, Germany). They were maintained with RPMI-1640 GlutaMAX medium supplemented with 10% inactivated FBS and 1% penicillin/streptomycin (Gibco, Thermofisher, Waltham, MA, USA). Each flask contained 12 mL of medium and was kept in a humidified incubator at 37 °C with 5% CO_2_. Cell lines had a mean passage number between 6–7 when obtaining the pellet (10 × 10^6^ cells) for DNA extraction.

### 4.3. DNA Extraction and Array Processing

DNA was extracted from 20 mg of frozen prefrontal cortex samples using QIAmp DNA Mini Kit (Qiagen, Hilden, Germany), with a DNA yield of 56–188 ng/μL in a final volume of 80 μL. DNA extraction from LCLs (10 × 10^6^ cells) was performed with AllPrep DNA/RNA/Protein Mini Kit (Qiagen), following the manufacturer’s protocol, with a DNA yield of 60–930 ng/μL in a final volume of 50 μL. Before performing the methylation studies, DNA integrity quality control was performed, and DNA samples were treated with RNaseA for 1 h at 45 °C.

A total of 600 ng of purified DNA was randomly distributed on a 96-well plate and processed using an EZ-96 DNA Methylation kit (Zymo Research Corp., Irvine, CA, USA), following the manufacturer’s recommendations for Infinium assays. Bisulfite-converted DNA was hybridized using an Infinium DNA MethylationEPIC BeadChip (Illumina Inc., San Diego, CA, USA). The efficiency of bisulfite conversion reaction was checked, with Illumina’s internal controls being optimal in all cases and without significant differences between sample groups.

Microarray data are available in the ArrayExpress database (http://www.ebi.ac.uk/arrayexpress, accessed on 1 January 2024) under accession number E-MTAB-11975.

### 4.4. Microarray Data and Biological Significance Analysis

Data analysis was performed using the statistical language R (https://www.r-project.org/, accessed on 1 January 2024) (version 4.1.0) and the following packages: Rfit (version 0.24.2), betareg (version 3.1-4), glmnet (version 4.1-1), IlluminaHumanMethylationEPICmanifest (version 0.3.0), NMF (version 0.23.0), Rtsne (version 0.15), pcaMethods (version 1.79.1), VennDiagram (version 1.6.20), and UpsetR (version 1.4.0).

After functional normalization, CpGs with a *p* value > 0.01 and those related to sexual chromosomes were filtered. A Beta regression model, with age and sex included as covariates, was applied to obtain differentially methylated positions (DMPs), and all *p*-values were adjusted according to the False Discovery Rate (FDR). Filters applied for further analyses of common DMPs between comparisons were: adjusted-*p* value < 0.05 and an absolute value of Beta difference > 0.25 for brain samples, and adjusted-*p* value < 0.01 and an absolute value of Beta difference > 0.35 in LCLs data; these filters were applied to obtain a manageable and similar number of DMPs in each comparison’s list. In addition to the Beta regression model, we performed an Elastic Net logistic regression [76]. This is a penalized linear regression model that allows the identification of a list of CpGs that may classify subjects to a diagnostic group. Sex and age were also included in the analyses as covariates. Selection of the regularization parameter was performed using 10-fold cross-validation with 200 repeats.

Venn diagrams (http://www.interactivenn.net/index.html, accessed on 1 January 2024) [77] and pseudo-Venn figures (UpsetR package in R) were created to visualize common genes between pairwise comparisons. Heatmaps were also produced, showing the top 10 DMPs with the highest Beta difference absolute value.

The biological significance analysis of the significative DMPs was performed by using Reactome Pathway Knowledge database (https://reactome.org/, accessed on 1 January 2024) and Gene Ontology (GO) resource with ShinyGO 0.76 tool (http://bioinformatics.sdstate.edu/go/, accessed on 1 January 2024) [78,79] in an over-representation analysis, using the following filters for Beta regression data: brain tissue, adjusted-*p* value < 0.05, and absolute value of Beta difference > 0.23, and for LCLs, adjusted-*p* value < 0.01 and absolute value of Beta difference > 0.32.

### 4.5. Pyrosequencing

Briefly, genomic DNA was bisulfite-converted as described above; then, polymerase chain reaction (PCR) was performed using standard conditions with biotinylated primers (primer sequences are shown in Supplemental Material S2). Pyrosequencing reactions and methylation quantification were performed using PyroMark Q24 System version 2.0.7 (Qiagen), using appropriate reagents and recommended protocols.

Statistical analysis was conducted with GraphPad Prism (version 8.0.2) using non-parametric tests.

### 4.6. Correlation between DNA Methylation Data and Gene Expression Array Data

We performed a correlation analysis of the Beta regression and Elastic Net results of each comparison with the expression level of all the genes from Clariom D array data from our previous study [20] using Pearson correlation coefficient (r), taking into account the closest 5 kb up- and downstream of each studied gene. Results show each tissue analyzed independently and using the following filters: an absolute value of *r* > 0.7, adjusted-*p* value < 0.05, and an absolute value of Beta difference > 0.1.

## Figures and Tables

**Table 1:** Correlation between CpGs obtained in the methylation analysis and genes found in the expression array.

	BRAIN				
Comparison	CpG	Gene	Correlation	Relation to Nearest Gene	Relation to CpG Island
PSEN1 vs. sEOAD	cg16550453	*TDRD1*	−0.8336	TSS200	Island
sFTD-Tau vs. CTRL	cg12150421	*KIF17*	−0.7075	Body	S_Shore
sFTD-TDP43 vs. CTRL	cg12150421	*KIF17*	−0.7075	Body	S_Shore
GRN vs. CTRL	cg24203376	*TDRD1*	−0.8060	TSS200	N_Shore
	cg05726248	*TESPA1*	−0.7070	TSS1500; ExonBnd; Body	0
	**LCLs**				
sEOAD vs. CTRL	cg17369694	*HLA.DRB5*	0.8304	3′UTR	0
	cg01341801	*HLA.DRB5*	0.8436	Body	N_Shore
	cg22730830	*PRSS21*	−0.8250	Body	Island
	cg01232511	*PRSS21*	−0.8564	Body	Island
PSEN1 vs. CTRL	cg09074040	*ANKDD1A*	0.7978	Body	0
PSEN1 vs. sEOAD	cg21817187	*SARM1*	0.7820	Body	N_Shore
MAPT vs. CTRL	cg17369694	*HLA.DRB5*	0.8304	3′UTR	0
	cg01341801	*HLA.DRB5*	0.8436	Body	N_Shore
	cg05072008	*FIGNL1*	0.7839	TSS1500	Island
	cg22730830	*PRSS21*	−0.8250	Body	Island
	cg01232511	*PRSS21*	−0.8564	Body	0
	cg25206919	*TRIM72*	0.8269	Body; 3′UTR	Island
GRN vs. CTRL	cg17369694	*HLA.DRB5*	0.8304	3′UTR	0
	cg01341801	*HLA.DRB5*	0.8436	Body	N_Shore
GRN vs. MAPT	cg05072008	*FIGNL1*	0.7839	TSS1500	Island
	cg20322685	*BAIAP2L1*	0.8185	Body	0

Comparisons which do not appear did not have any significative result. For each CpG, it is also shown its relation to the nearest gene and its relative position with respect to a CpG island. Filters applied: adjusted-*p* value < 0.05; absolute value of Beta difference Beta difference > [0.1]; correlation coefficient (*r*) > [0.7]. Abbreviations: CTRL, healthy controls; sEOAD, sporadic early-onset Alzheimer’s disease; PSEN1, autosomal dominant Alzheimer’s disease due to mutation in *PSEN1*; MAPT, GRN, familial frontotemporal dementia due to mutation in *MAPT* or *GRN*; sFTD-Tau, sporadic frontotemporal dementia with accumulation of tau; sFTD-TDP43, sporadic frontotemporal dementia with accumulation of TDP43; LCLs, lymphoblastoid cell lines.

**Table 2:** Demographics of each group included.

		Samples (*n* = 64)	Sex; Female/Male (% Female)	Age at Sampling	Age at Onset	Years since Onset	Post Mortem Delay (h)	Passage Number
BRAIN (*n* = 40)	CTRL	5	2/3 (40)	54.0 ± 19.3	//	//	11.2 ± 6.5	//
	sEOAD	5	3/2 (60)	67.0 ± 4.4	55.6 ± 4.4	11.5 ± 5.6	7.3 ± 2.6	//
	PSEN1	5	3/2 (60)	54.0 ± 3.7	41.8 ± 7.2	12.2 ± 7.8	9.6 ± 5.7	//
	MAPT	5	1/4 (20)	60.2 ± 7.4	52.8 ± 4.7	7.4 ± 3.8	11.4 ± 3.5	//
	GRN	5	3/2 (60)	69.6 ± 5.2	62.2 ± 6.7	7.4 ± 2.1	15.6 ± 4.4	//
	C9orf72	5	2/3 (40)	71.4 ± 14.8	60.0 ± 14.9	9.3 ± 1.7	7.4 ± 3.3	//
	sFTD-Tau	5	1/4 (20)	70.0 ± 6.6	60.4 ± 5.9	9.6 ± 3.6	8.3 ± 5.7	//
	sFTD-TDP43	5	2/3 (40)	71.2 ± 9.4	57.4 ± 7.1	13.8 ± 3.6	11.8 ± 1.8	//
LCLs (*n* = 24)	CTRL	5	2/3 (40)	48.8 ± 10.3	//	//	//	6.6 ± 0.5
	sEOAD	5	2/3 (40)	59.8 ± 4.7	55.4 ± 3.2	4.4 ± 1.8	//	5.6 ± 1.5
	PSEN1	6	2/4 (33)	49.3 ± 6.6	44.8 ± 7.4	4.5 ± 2.0	//	5.2 ± 0.8
	MAPT	3	2/1 (67)	67.0 ± 11.7	59.7 ± 9.5	7.3 ± 7.6	//	6.0 ± 2.0
	GRN	5	5/0 (100)	59.0 ± 3.3	56.0 ± 2.5	3.0 ± 1.3	//	5.8 ± 2.0

Mean values (±SD) are represented for each subject group. Abbreviations: CTRL, healthy controls; sEOAD, sporadic early-onset Alzheimer’s disease; PSEN1, autosomal dominant Alzheimer’s disease due to mutation in *PSEN1*; MAPT, GRN, C9orf72, familial frontotemporal dementia due to mutation in *MAPT*, *GRN* or *C9orf72*; sFTD-Tau, sporadic frontotemporal dementia with accumulation of tau; sFTD-TDP43, sporadic frontotemporal dementia with accumulation of TDP43; LCLs, lymphoblastoid cell lines.

## Data Availability

Microarray data are available in the ArrayExpress database (http://www.ebi.ac.uk/arrayexpress, accessed on 1 January 2024) under accession number E-MTAB-11975.

## Supplementary Materials

The following supporting information can be downloaded at https://www.mdpi.com/article/10.3390/ijms25105445/s1.
