# Supplementary material for: Genome-Wide DNA Methylation in Early-Onset-Dementia Patients Brain Tissue and Lymphoblastoid Cell Lines
Source: Int J Mol Sci. 2024 May 16;25(10):5445. doi: 10.3390/ijms25105445 (PMC11121630; doi:10.3390/ijms25105445)
Supplement: Supplementary file 1 [file ijms-25-05445-s001.zip › Supplemental material S1. MutationsList.pdf]

Additional file S1. List of all mutations included for each genetic group in both tissues.

Brain			LCLs		
GRN	***MAPT***	***PSEN1***	***GRN***	***MAPT***	***PSEN1***
p.Q454AfsX54	p.P301L	p.L286P	p.C366fsX1	p.P301L	p.L282R
c.709-1G>A	p.P301L	p.M139T	p.C366fsX1	p.P301L	p.I439S
c.709-1G>A	p.P301L	p.P264L	p.C366fsX1	p.P397S	p.G206D
p.A303AfsX57	p.P301L	p.M139T	p.Q257PfsX27		p.L286P
c.708+6_+9delTGAG	p.P301L	p.M139T	p.C366fsX1		p.T116I
					p.K239N

Abbreviations: GRN, MAPT, familial frontotemporal dementia caused by mutation in *GRN* or *MAPT*; PSEN1, autosomal dominant Alzheimer's disease caused by mutation in *PSEN1*; LCLs, lymphoblastoid cell lines.
