# Supplementary material for: Genome-Wide DNA Methylation in Early-Onset-Dementia Patients Brain Tissue and Lymphoblastoid Cell Lines
Source: Int J Mol Sci. 2024 May 16;25(10):5445. doi: 10.3390/ijms25105445 (PMC11121630; doi:10.3390/ijms25105445)
Supplement: Supplementary file 1 [file ijms-25-05445-s001.zip › Supplemental material S10. Common CpGs_AD+FTD.pdf]

Additional file S10. Common differentially methylated CpGs (DMPs) found between all patients vs. control comparisons performed.

Comparisons	Common DMPs list (Brain)
sEOAD vs. CTRL<br>PSEN1 vs. CTRL<br>sFTD-Tau vs. CTRL<br>sFTD-TDP43 vs. CTRL<br>MAPT vs. CTRL<br>GRN vs. CTRL<br>C9orf72 vs. CTRL	cg17925226
sEOAD vs. CTRL<br>PSEN1 vs. CTRL<br>sFTD-TDP43 vs. CTRL<br>MAPT vs. CTRL<br>GRN vs. CTRL<br>C9orf72 vs. CTRL	cg06354054
PSEN1 vs. CTRL<br>sFTD-Tau vs. CTRL<br>sFTD-TDP43 vs. CTRL<br>MAPT vs. CTRL<br>GRN vs. CTRL<br>C9orf72 vs. CTRL	cg07902519
sEOAD vs. CTRL<br>PSEN1 vs. CTRL<br>sFTD-TDP43 vs. CTRL<br>MAPT vs. CTRL<br>GRN vs. CTRL	cg02945674
sEOAD vs. CTRL<br>PSEN1 vs. CTRL<br>sFTD-TDP43 vs. CTRL<br>MAPT vs. CTRL<br>C9orf72 vs. CTRL	cg14866535

sEOAD vs. CTRL<br>PSEN1 vs. CTRL<br>sFTD-TDP43 vs. CTRL<br>GRN vs. CTRL<br>C9orf72 vs. CTRL	cg05232042
PSEN1 vs. CTRL<br>sFTD-Tau vs. CTRL<br>sFTD-TDP43 vs. CTRL<br>GRN vs. CTRL<br>C9orf72 vs. CTRL	cg10737404
sEOAD vs. CTRL<br>PSEN1 vs. CTRL<br>sFTD-TDP43 vs. CTRL<br>C9orf72 vs. CTRL	cg05966078
sEOAD vs. CTRL<br>sFTD-Tau vs. CTRL<br>sFTD-TDP43 vs. CTRL<br>GRN vs. CTRL	cg22481673; cg00892228
sEOAD vs. CTRL<br>sFTD-TDP43 vs. CTRL<br>MAPT vs. CTRL<br>C9orf72 vs. CTRL	cg22955899
sEOAD vs. CTRL<br>sFTD-TDP43 vs. CTRL<br>GRN vs. CTRL<br>C9orf72 vs. CTRL	cg07319199
sEOAD vs. CTRL<br>MAPT vs. CTRL<br>GRN vs. CTRL<br>C9orf72 vs. CTRL	cg15292356; cg13521643
PSEN1 vs. CTRL<br>sFTD-Tau vs. CTRL<br>sFTD-TDP43 vs. CTRL<br>GRN vs. CTRL	cg11969330; cg05834845

PSEN1 vs. CTRL<br>sFTD-Tau vs. CTRL<br>MAPT vs. CTRL<br>C9orf72 vs. CTRL	cg09061753
PSEN1 vs. CTRL<br>sFTD-Tau vs. CTRL<br>GRN vs. CTRL<br>C9orf72 vs. CTRL	cg11054397
PSEN1 vs. CTRL<br>sFTD-TDP43 vs. CTRL<br>MAPT vs. CTRL<br>C9orf72 vs. CTRL	cg00899976
PSEN1 vs. CTRL<br>sFTD-TDP43 vs. CTRL<br>GRN vs. CTRL<br>C9orf72 vs. CTRL	cg07472332
PSEN1 vs. CTRL<br>MAPT vs. CTRL<br>GRN vs. CTRL<br>C9orf72 vs. CTRL	cg15950547
sEOAD vs. CTRL<br>PSEN1 vs. CTRL<br>sFTD-Tau vs. CTRL	cg08522473
sEOAD vs. CTRL<br>PSEN1 vs. CTRL<br>C9orf72 vs. CTRL	cg01059116
sEOAD vs. CTRL<br>sFTD-Tau vs. CTRL<br>sFTD-TDP43 vs. CTRL	cg24996718; cg04034998
sEOAD vs. CTRL<br>sFTD-TDP43 vs. CTRL<br>MAPT vs. CTRL	cg26983017

sEOAD vs. CTRL<br>sFTD-TDP43 vs. CTRL<br>GRN vs. CTRL	cg20017683
sEOAD vs. CTRL<br>sFTD-TDP43 vs. CTRL<br>C9orf72 vs. CTRL	cg08269402
sEOAD vs. CTRL<br>GRN vs. CTRL<br>C9orf72 vs. CTRL	cg07568443
PSEN1 vs. CTRL<br>sFTD-Tau vs. CTRL<br>MAPT vs. CTRL	cg00403716; cg22244330; cg20565764
PSEN1 vs. CTRL<br>sFTD-Tau vs. CTRL<br>GRN vs. CTRL	cg06508795
PSEN1 vs. CTRL<br>sFTD-Tau vs. CTRL<br>C9orf72 vs. CTRL	cg19389973
PSEN1 vs. CTRL<br>sFTD-TDP43 vs. CTRL<br>GRN vs. CTRL	cg21102584
PSEN1 vs. CTRL<br>MAPT vs. CTRL<br>GRN vs. CTRL	cg20012315; cg15421137; cg17370981
PSEN1 vs. CTRL<br>GRN vs. CTRL<br>C9orf72 vs. CTRL	cg12622172; cg13337899; cg00713204
sEOAD vs. CTRL<br>PSEN1 vs. CTRL	cg14827740
sEOAD vs. CTRL<br>sFTD-Tau vs. CTRL	cg04892437 cg16816400

sEOAD vs. CTRL<br>sFTD-TDP43 vs. CTRL	cg23957735; cg08283200; cg14114910; cg18561199; cg08600378; cg05338009
sEOAD vs. CTRL<br>MAPT vs. CTRL	cg11986743; cg11973981
sEOAD vs. CTRL<br>GRN vs. CTRL	cg00125893; cg09829645
sEOAD vs. CTRL<br>C9orf72 vs. CTRL	cg11008123; cg20215438; cg10759817; cg00012123
PSEN1 vs. CTRL<br>sFTD-Tau vs. CTRL	cg01483656; cg17056069; cg19882830; cg09352518; cg23350716; cg01326874; cg05867245; cg23333490
PSEN1 vs. CTRL<br>sFTD-TDP43 vs. CTRL	cg15399577; cg13989295; cg02246922; cg01081438
PSEN1 vs. CTRL<br>MAPT vs. CTRL	cg20406175; cg10361788; cg00866176
PSEN1 vs. CTRL<br>GRN vs. CTRL	cg10834425; cg22024931; cg10773971; cg11823448
PSEN1 vs. CTRL<br>C9orf72 vs. CTRL	cg01462799; cg04026937

Comparisons	Common DMPs list (LCLs)
sEOAD vs. CTRL<br>PSEN1 vs. CTRL<br>GRN vs. CTRL	cg25004725
sEOAD vs. CTRL<br>MAPT vs. CTRL	cg15897921; cg25641126; cg08709228; cg15768146; cg07078430; cg02691373
sEOAD vs. CTRL<br>GRN vs. CTRL	cg04705480
PSEN1 vs. CTRL<br>MAPT vs. CTRL	cg18197594; cg05082563; cg24923430; cg11954332; cg02505002; cg11078654; cg10501835
PSEN1 vs. CTRL<br>GRN vs. CTRL	cg04797323; cg03013609; cg20868217; cg14252149; cg14323910

Filters applied: in brain adjusted-p value <0.05 and absolute value of Beta difference >0.25; in LCLs adjuted-p value <0.01 and absolute value of Beta difference >0.35. Abbreviations: CTRL, healthy controls; sEOAD, sporadic early-onset Alzheimer's disease; PSEN1, autosomal dominant Alzheimer's disease caused by mutation in *PSEN1* gene; MAPT, GRN, C9orf72, familial frontotemporal dementia caused by mutation in *MAPT*, *GRN* or *C9orf72* genes; sFTD-Tau, sporadic frontotemporal dementia with tau deposits; sFTD-TDP43, sporadic frontotemporal dementia with TDP43 deposits; LCLs, lymphoblastoid cell lines.
