# Supplementary material for: Genome-Wide DNA Methylation in Early-Onset-Dementia Patients Brain Tissue and Lymphoblastoid Cell Lines
Source: Int J Mol Sci. 2024 May 16;25(10):5445. doi: 10.3390/ijms25105445 (PMC11121630; doi:10.3390/ijms25105445)
Supplement: Supplementary file 1 [file ijms-25-05445-s001.zip › Supplemental material S11. ElasticNet.pdf]

Additional file S11.

A) Equations for discriminating between the different compared groups using the combination of CpGs resulted from Elastic Net logistic regressions. The provided equations are for the linear predictor. To get the final probability, the following general formula has to be used: $Prob(altered) = \frac{e^{LP}}{1+e^{LP}}$

Comparisons	Elastic Net results (Brain)
sEOAD vs. CTRL	-2.899 + 0.173*cg01059116 + 0.73*cg01371348 + 0.316*cg05451400 + 0.378*cg19921085 + 0.001*cg01823663 + 0.469*cg24766821 + 0.031*cg10360403 + 0.446*cg10881242 + -0.066*cg02458000 + 0.327*cg18912520 + 0.332*cg15390122 + 0.038*cg24084481 + -0.393*cg15684290 + 0.616*cg11809339 + 0.486*cg23968456 + 0.556*cg02467451 + 0.432*cg18748888
PSEN1 vs. CTRL	-0.631 + 0.005*cg04083553 + 0.121*cg07622001 + 0.063*cg26961808 + -0.032*cg11420142 + 0.227*cg07729393 + 0.094*cg23914904 + 0.343*cg16162172
sFTD-Tau vs. CTRL	-4.46 + 0.122*cg20987072 + 0.003*cg01048749 + 0.441*cg17600743 + -0.183*cg06264882 + 0.521*cg15066503 + 1.744*cg27335600 + 0.347*cg00597607 + 0.057*cg11969330 + 0.524*cg01302593 + 0.905*cg21010220 + 0.212*cg09715499 + 0.007*cg05017760 + 0.552*cg02780400 + 2.352*cg03602823 + 1.391*cg08288469 + 0.221*cg17217228 + 0*cg20889818 + -0.181*cg07884306 + 0.163*cg15784814 + 0.032*cg03476323
sFTD-TDP43 vs. CTRL	-3.261 + 0.006*cg27197651 + 0.375*cg04553810 + 1.538*cg05909164 + 0.374*cg16668359 + 0.159*cg16619576 + 0.007*cg13176012 + 0.224*cg19244790 + 0.626*cg16994200 + 0.044*cg02958670 + 0.055*cg12190504 + 0.148*cg01533585 + 0.011*cg12029281 + 0.001*cg23639368 + 0.081*cg01867538 + -0.132*cg06073220 + 4.548*cg18285951 + -0.164*cg10831642 + 0.844*cg26419957 + 0.589*cg03898385 + 0.253*cg16359034 + 0.166*cg04891961 + 0.345*cg27583483 + 0.217*cg18748888 + 0.26*cg09105928 + 0.136*cg04203646 + 0.347*cg25652610 + 0.14*cg03994651 + -0.093*cg24641125
MAPT vs. CTRL	-1.594 + 0.016*cg16970559 + 0.024*cg26205842 + 0.02*cg03902905 + 0.066*cg02958670 + 0.23*cg09288273 + 0.015*cg06495631 + 0.019*cg01630199 + 0.034*cg22395253 + 0.855*cg06897306 + 0.363*cg14486338 + 0.001*cg11673283 + 0.002*cg24037820 + 0.011*cg04076403 + 0.527*cg03928367 + 0.061*cg11316868 + 0.024*cg02571448
GRN vs. CTRL	-5.918 + 0.026*cg08872579 + 0.335*cg06577951 + 0.02*cg06930255 + 0.014*cg04391718 + 0.069*cg02958670 + 0.013*cg10987720 + 0.017*cg16479474 + 0.787*cg16565902 + 2.981*cg16219283 + 0.141*cg14168080 + 0.068*cg01354455 + 0.406*cg19866040 + 0.009*cg10833066 + 0.153*cg17217228 + 0.125*cg14870958 + 1.084*cg05189517 + 0.283*cg09155905 + 0.001*cg27583483 + 0.39*cg12582138 + 7.375*cg25120928 + 0.491*cg15302968 + 2.379*cg26026416 + 0.145*cg17091610 + 0.167*cg17848838
C9orf72 vs. CTRL	-4.703 + 0.863*cg05617030 + 0.069*cg21577049 + 1.963*cg08551428 + 0.125*cg27288240 + 0.078*cg25654697 + 1.838*cg10072502 + -0.143*cg21211688 + 0.006*cg01354455 + 2.446*cg19219920 + 0.994*cg25161474 + 0.084*cg25045882 + 2.248*cg21009935 + 0.404*cg03588513 + 2.669*cg12177023 + 0.202*cg07821960 + 9.299*cg02792677 + 0.327*cg23504701
**Comparisons**	**Elastic Net results (LCLs)**
sEOAD vs. CTRL	15.633 + -0.008*cg01546084 + 12.258*cg04124260 + -0.144*cg24591105 + -0.022*cg14300132 + -0.068*cg03589296 + -1.682*cg16077818 + -0.119*cg14014369 + -1.358*cg13918646 + -0.046*cg10640830 + -1.724*cg04167833 + -0.497*cg15147693 + -0.027*cg20883227 + -0.003*cg01801101 + -0.511*cg10945855 + -0.403*cg10220850 + -0.317*cg10515852 + -5.04*cg10810860 + -0.173*cg14651896 + -0.146*cg20201177 + 8.625*cg07259717 + -2.776*cg00051979 + -0.346*cg16192621 + -0.165*cg02523018 + -0.885*cg17497052 + 0.321*cg11831988 + -1.125*cg10774305 + -0.038*cg27002522 + -0.001*cg04315470 + -0.153*cg10298855 + 0.027*cg15302765 + 6.849*cg09448951 + -0.373*cg23414361 + -1.846*cg03012230 + -0.371*cg02375258 + -0.016*cg01695406 + -0.814*cg08496404 + -4.892*cg15335837 + 0.033*cg20126498

PSEN1 vs. CTRL	0.271 + 0.609*cg04724058 + -0.066*cg09810149 + -0.305*cg09391823 + 0.565*cg04837898 + 5.216*cg02652511 + 2.768*cg22024370 + -0.005*cg24558204 + -0.013*cg11078654 + 1.072*cg05112824 + -0.422*cg01459283 + 0.022*cg21394561 + -0.082*cg12460598 + 0.167*cg13803003 + 4.314*cg03579738 + -0.194*cg12695921
MAPT vs. CTRL	0.695 + -0.025*cg21787209 + 0.012*cg03544800 + -0.012*cg10214933 + -0.129*cg00613416 + -0.009*cg21580594 + 0.029*cg25234732 + -0.015*cg02153747 + -0.009*cg26919731 + -0.036*cg22086536 + -0.05*cg01459283 + -0.007*cg25249386 + -0.01*cg02011185 + -0.028*cg07203494 + 0.276*cg12249207
GRN vs. CTRL	8.307 + 0.061*cg18138172 + 0.216*cg04101267 + 5.291*cg02260687 + 0.032*cg06160440 + 2.457*cg09040434 + 0.046*cg02458039 + -2.626*cg01368406 + 0.849*cg24886176 + 0*cg21507332 + -0.34*cg11503661 + -0.436*cg02310273 + 0.272*cg25137613 + 0.921*cg18065337 + -0.483*cg23529139 + -3.556*cg06656551 + 17.223*cg15956008 + 0.551*cg04090147 + 2.516*cg14708076 + -0.16*cg04960128 + -0.355*cg16345566 + -0.713*cg03768479 + -0.536*cg00815484 + 0.185*cg08919780 + 3.406*cg09866690 + 0*cg02603686 + 2.277*cg03637614 + 2.357*cg21906414 + -0.651*cg14651896 + 0.294*cg01796367 + 0*cg10749860 + 0.002*cg18129327 + -0.363*cg16344810 + -0.293*cg14499074 + 0.002*cg13760807 + 0.005*cg00770636 + 2.374*cg20492200 + -0.995*cg03822098 + 0.492*cg23272632 + 0.838*cg07943633 + -0.357*cg01374787 + 2.598*cg07359369 + 0.271*cg17381632 + 0.002*cg17444839 + 0*cg27002699 + 0.954*cg16880783 + 7.337*cg22492273 + -1.383*cg13677144 + 6.038*cg16400971 + 0.971*cg03304299 + -0.882*cg18655348 + 1.457*cg19348733 + -0.248*cg11425737

Abbreviations: CTRL, healthy controls; sEOAD, sporadic early-onset Alzheimer's disease; PSEN1, autosomal dominant Alzheimer's disease caused by mutation in *PSEN1*; MAPT, GRN, C9orf72, familial frontotemporal dementia caused by mutation in *MAPT*, *GRN* or *C9orf72*; sFTD-Tau, sporadic frontotemporal dementia with tau deposits; sFTD-TDP43, sporadic frontotemporal dementia with TDP43 deposits; LCLs, lymphoblastoid cell lines; LP, linear predictor.

B) CpGs resulted from Elastic Net logistic regression (wtihout equations)

**Comparisons**	**Elastic Net results (Brain)**
sEOAD vs. CTRL	cg01059116; cg01371348; cg05451400; cg19921085; cg01823663; cg24766821; cg10360403; cg10881242; cg02458000; cg18912520; cg15390122; cg24084481; cg15684290; cg11809339; cg23968456; cg02467451; cg18748888
PSEN1 vs. CTRL	cg04083553; cg07622001; cg26961808; cg11420142; cg07729393; cg23914904; cg16162172
sFTD-Tau vs. CTRL	cg00597607; cg01048749; cg01302593; cg02780400; cg03476323; cg03602823; cg05017760; cg06264882; cg07884306; cg08288469; cg09715499; cg11969330; cg15066503; cg15784814; cg17217228; cg17600743; cg20889818; cg20987072; cg21010220; cg27335600
sFTD-TDP43 vs. CTRL	cg01533585; cg01867538; cg02958670; cg03898385; cg03994651; cg04203646; cg04553810; cg04891961; cg05909164; cg06073220; cg09105928; cg10831642; cg12029281; cg12190504; cg13176012; cg16359034; cg16619576; cg16668359; cg16994200; cg18285951; cg18748888; cg19244790; cg23639368; cg24641125; cg25652610; cg26419957; cg27197651; cg27583483

MAPT vs. CTRL	cg01630199; cg02571448; cg02958670; cg03902905; cg03928367; cg04076403; cg06495631; cg06897306; cg09288273; cg11316868; cg11673283; cg14486338; cg16970559; cg22395253; cg24037820; cg26205842
GRN vs. CTRL	cg01354455; cg02958670; cg04391718; cg05189517; cg06577951; cg06930255; cg08872579; cg09155905; cg10833066; cg10987720; cg12582138; cg14168080; cg14870958; cg15302968; cg16219283; cg16479474; cg16565902; cg17091610; cg17217228; cg17848838; cg19866040; cg25120928; cg26026416; cg27583483
C9orf72 vs. CTRL	cg01354455; cg02792677; cg03588513; cg05617030; cg07821960; cg08551428; cg10072502; cg12177023; cg19219920; cg21009935; cg21211688; cg21577049; cg23504701; cg25045882; cg25161474; cg25654697; cg27288240
**Comparisons**	**Elastic Net results (LCLs)**
sEOAD vs. CTRL	cg01546084; cg04124260; cg24591105; cg14300132; cg03589296; cg16077818; cg14014369; cg13918646; cg10640830; cg04167833; cg15147693; cg20883227; cg01801101; cg10945855; cg10220850; cg10515852; cg10810860; cg14651896; cg20201177; cg07259717; cg00051979; cg16192621; cg02523018; cg17497052; cg11831988; cg10774305; cg27002522; cg04315470; cg10298855; cg15302765; cg09448951; cg23414361; cg03012230; cg02375258; cg01695406; cg08496404; cg15335837; cg20126498
PSEN1 vs. CTRL	cg04724058; cg09810149; cg09391823; cg04837898; cg02652511; cg22024370; cg24558204; cg11078654; cg05112824; cg01459283; cg21394561; cg12460598; cg13803003; cg03579738; cg12695921
MAPT vs. CTRL	cg00613416; cg01459283; cg02011185; cg02153747; cg03544800; cg07203494; cg10214933; cg12249207; cg21580594; cg21787209; cg22086536; cg25234732; cg25249386; cg26919731
GRN vs. CTRL	cg00770636; cg00815484; cg01368406; cg01374787; cg01796367; cg02260687; cg02310273; cg02458039; cg02603686; cg03304299; cg03637614; cg03768479; cg03822098; cg04090147; cg04101267; cg04960128; cg06160440; cg06656551; cg07359369; cg07943633; cg08919780; cg09040434; cg09866690; cg10749860; cg11425737; cg11503661; cg13677144; cg13760807; cg14499074; cg14651896; cg14708076; cg15956008; cg16344810; cg16345566; cg16400971; cg16880783; cg17381632; cg17444839; cg18065337; cg18129327; cg18138172; cg18655348; cg19348733; cg20492200; cg21507332; cg21906414; cg22492273; cg23272632; cg23529139; cg24886176; cg25137613; cg27002699

Abbreviations: CTRL, healthy controls; sEOAD, sporadic early-onset Alzheimer's disease; PSEN1, autosomal dominant Alzheimer's disease caused by mutation in *PSEN1*; MAPT, GRN, C9orf72, familial frontotemporal dementia caused by mutation in *MAPT*, *GRN* or *C9orf72*; sFTD-Tau, sporadic frontotemporal dementia with tau deposits; sFTD-TDP43, sporadic frontotemporal dementia with TDP43 deposits; LCLs, lymphoblastoid cell lines.
