# Supplementary material for: Genome-Wide DNA Methylation in Early-Onset-Dementia Patients Brain Tissue and Lymphoblastoid Cell Lines
Source: Int J Mol Sci. 2024 May 16;25(10):5445. doi: 10.3390/ijms25105445 (PMC11121630; doi:10.3390/ijms25105445)
Supplement: Supplementary file 1 [file ijms-25-05445-s001.zip › Supplemental material S2. Pyrosequence primers + validation results.pdf]

Additional file S2.

a) Complete list of primers employed in the validation analysis.

Gene	Oligo Name	Sequence
TUBAL3	TUBAL3-F	AGTAGTTGTGTTGGGTTTGAATA
	Bio-TUBAL3-R	TACTCCTTCCTTTTCTCTACCAATACTA
	TUBAL3-Seq	GTGTTGGGTTTGAATAAT
ABCA1	ABCA1-F	AGGTGGAGGTTGTAGTGA
	Bio-ABCA1-R	CTCCCCTACAAATAAACATTTAAAATC
	ABCA1-Seq	TGTTTAGGGTTTATTATAGGT
SRPK2	SRPK2-F	ATGGGAAGATTTATAGGTGATGAGAAAGA
	Bio-SRPK2-R	TTTCAATCCCTTCCCTCCAATTTT
	SRPK2-Seq	ATGGGTTTTTAATTTTTATAAAT

b) Results for pyrosequencing validation:

Genes	CpG	Comparisons	p value
TUBAL3	cg10890644	sFTD-TDP43 vs sFTD-Tau	0.0079
ABCA1	cg02945674	sEOAD vs CTRL	0.0204
		PSEN1 vs CTRL	
		PSEN1 vs sEOAD	

For each gene selected, the related CpG and significative comparison in which it was found are shown, as well as the p-value of the beta regression model performed. Abbreviations: CTRL, healthy controls; sEOAD, sporadic early-onset Alzheimer's disease; PSEN1, autosomal dominant Alzheimer's disease due to mutation in *PSEN1*; sFTD-Tau, sporadic frontotemporal dementia with accumulation of tau; sFTD-TDP43, sporadic frontotemporal dementia with accumulation of TDP43.
