# Supplementary material for: Genome-Wide DNA Methylation in Early-Onset-Dementia Patients Brain Tissue and Lymphoblastoid Cell Lines
Source: Int J Mol Sci. 2024 May 16;25(10):5445. doi: 10.3390/ijms25105445 (PMC11121630; doi:10.3390/ijms25105445)
Supplement: Supplementary file 1 [file ijms-25-05445-s001.zip › Supplemental material S3. Number_CpGs.pdf]

Additional file S3. Number of differentially methylated CpGs (DMPs) found in each comparison and region.

Comparison	Total	HYPER met.	HYPO met.	CpG Island	HYPER met.	HYPO met.	N_Shore	HYPER met.	HYPO met.	S_Shore	HYPER met.	HYPO met.	N_Shelf	HYPER met.	HYPO met.	S_Shelf	HYPER met.	HYPO met.
**BRAIN**																		
sEOAD vs. CTRL	29133	25026	4107	5185	3025	2160	2877	2557	320	2383	2083	300	1189	1106	83	1167	1100	67
PSEN1 vs. CTRL	22857	19200	3657	4306	3205	1101	2381	2008	373	2159	1790	369	900	751	149	752	629	123
PSEN1 vs. sEOAD	102488	71272	31216	21990	14596	7394	9573	6674	2895	8099	5699	2396	3502	2500	1002	3382	2380	1000
sFTD-Tau vs. CTRL	156601	143485	13116	18871	13204	5667	12667	11470	1197	10683	9617	1066	6048	5692	356	5700	5398	302
sFTD-TDP43 vs. CTRL	316263	288975	27288	40090	29833	10257	28894	26629	2265	24354	22429	1925	12454	11628	826	11585	10856	729
sFTD-TDP43 vs. sFTD-Tau	14146	11837	2309	2892	2088	804	1295	989	306	1106	874	232	546	492	54	527	460	67
MAPT vs. CTRL	78204	71196	7008	16698	12116	4582	8925	8472	453	7612	7243	369	3489	3380	109	3297	3203	94
GRN vs. CTRL	173621	163815	9806	29787	23409	6378	15060	14270	790	12589	11983	606	6985	6801	184	6528	6385	143
C9orf72 vs. CTRL	80779	73706	7073	16913	13043	3870	8198	7527	671	6815	6247	568	3092	2927	165	2878	2753	125
**LCLs**																		
sEOAD vs. CTRL	43722	29038	14684	7723	3576	4147	5148	3076	2072	4294	2669	1625	1608	1231	377	1520	1151	369
PSEN1 vs. CTRL	42020	29146	12874	10195	2613	7582	3761	2477	1284	3176	2106	1070	1634	1485	149	1520	1378	142
MAPT vs. CTRL	121097	99020	22077	24448	16248	8200	12448	9791	2657	10661	8488	2173	4112	3540	572	3911	3359	552
GRN vs. CTRL	64514	31182	33332	23611	3014	20597	6339	3005	3334	5310	2563	2747	2045	1658	387	1867	1519	348
PSEN1 vs. sEOAD	33880	14606	19274	6348	2820	3528	3896	1822	2074	3352	1604	1748	1239	544	695	1082	446	636
GRN vs. MAPT	46790	11667	35123	15838	1892	13956	5988	1261	4727	5164	1174	3990	1294	545	749	1162	540	622

According to the relative position to the nearest CpG island, two different regions exist: Shores (2Kb from islands) and shelves (4Kb from islands). At the same time, each region may be closer to the 5' end (N region) or to the 3' end (S region). In comparisons between patients and controls, hyper or hypomethylation refers to the patients' group. Between two groups of patients, it refers to the second group of the comparison. Filters applied: adjusted-p value <0.05. Abbreviations: CTRL, healthy controls; sEOAD, sporadic early-onset Alzheimer's disease; PSEN1, autosomal dominant Alzheimer's disease caused by mutation in *PSEN1*; MAPT, GRN, C9orf72, familial frontotemporal dementia caused by mutation in *MAPT*, *GRN* or *C9orf72*; sFTD-Tau, sporadic frontotemporal dementia with tau deposits; sFTD-TDP43, sporadic frontotemporal dementia with TDP43 deposits; LCLs, lymphoblastoid cell lines.
