# Supplementary material for: Genome-Wide DNA Methylation in Early-Onset-Dementia Patients Brain Tissue and Lymphoblastoid Cell Lines
Source: Int J Mol Sci. 2024 May 16;25(10):5445. doi: 10.3390/ijms25105445 (PMC11121630; doi:10.3390/ijms25105445)
Supplement: Supplementary file 1 [file ijms-25-05445-s001.zip › Supplemental material S4. DMPs_Causative genes.pdf]

Additional file S4. Dementia causative genes and associated CpGs found in the methylation array.

BRAIN					
**Gene**	**CpG**	**Beta Difference**	**Adj.pval**	**Relation to nearest gene**	**Relation to CpG Island**
PSEN1	cg05385100	0.0483	0.0015	3'UTR	--
MAPT	cg01640727	0.1318	0.0017	5'UTR	S_Shelf
	cg01934064	0.1533	0.0000	Body	S_Shelf
	cg02159926	0.0885	0.0277	5'UTR	--
	cg02228913	0.1383	0.0005	Body	N_Shelf
	cg05396503	0.0596	0.0398	Body	--
	cg07368061	0.0954	0.0000	Body	--
	cg13773723	-0.0083	0.0000	TSS1500; 1stExon; Body; 5'UTR	Island
	cg14431592	0.0920	0.0277	TSS200; Body	--
	cg14888901	0.0890	0.0048	Body	--
	cg15682851	0.0270	0.0013	TSS200; Body	--
	cg16520312	0.0360	0.0140	TSS1500; Body	Island
	cg18228076	0.1423	0.0005	5'UTR	--
	cg18554795	0.0645	0.0011	TSS1500; Body	--
	cg19108736	0.1025	0.0065	5'UTR	S_Shelf
	cg20099416	-0.0054	0.0172	1stExon; 5'UTR; Body; TSS1500	Island
	cg20265358	0.0735	0.0110	Body	Island
	cg20750326	0.0675	0.0071	5'UTR	--
	cg21705961	0.0465	0.0274	Body	S_Shore
	cg24677220	0.0403	0.0000	Body; TSS1500	--
	cg24801230	0.2980	0.0000	5'UTR	S_Shelf
GRN	cg07777378	0.0026	0.0150	TSS200	--
	cg21689086	0.0065	0.0170	1stExon; 5'UTR	--

	cg23322957	0.0773	0.0083	5'UTR	--
	cg23570245	0.1123	0.0055	5'UTR	--
C9orf72	cg01589155	0.0333	0.0016	5'UTR; TSS200	S_Shore
	cg13958452	0.0574	0.0011	5'UTR	N_Shore
**LYMPHOBLASTOID CELL LINES**					
PSEN1	cg06211357	-0.0032	0.0026	TSS200	Island
	cg24216535	0.0536	0.0068	Body	--
	cg26376566	-0.0065	0.0013	5'UTR	Island
MAPT	cg02159926	-0.0192	0.0025	5'UTR	--
	cg04601362	0.1520	0.0000	5'UTR	--
	cg05533539	-0.1570	0.0442	3'UTR	--
	cg05721485	0.1209	0.0243	Body	--
	cg10780632	-0.0224	0.0383	5'UTR; TSS1500; Body	Island
	cg11909912	-0.0217	0.0043	5'UTR; Body	Island
	cg13100502	-0.0460	0.0192	Body	--
	cg13137533	-0.0191	0.0176	5'UTR; TSS1500; Body	Island
	cg13773723	-0.0132	0.0000	TSS1500; 1stExon; Body; 5'UTR	Island
	cg15617032	0.1814	0.0010	5'UTR	--
	cg15739383	0.0925	0.0267	5'UTR	--
	cg20265358	0.1376	0.0005	Body	Island
	cg23926235	0.0098	0.0101	5'UTR	N_Shelf
	cg25885883	0.1412	0.0048	TSS1500; Body	--
GRN	cg17101358	-0.0074	0.0079	5'UTR; 1stExon	--
	cg19107120	-0.0499	0.0014	3'UTR	N_Shore

For each of the CpGs found, the Beta difference, adjusted-p value and their relative positions are shown. Filters applied: adjusted-p value <0.05.
