# Supplementary material for: Genome-Wide DNA Methylation in Early-Onset-Dementia Patients Brain Tissue and Lymphoblastoid Cell Lines
Source: Int J Mol Sci. 2024 May 16;25(10):5445. doi: 10.3390/ijms25105445 (PMC11121630; doi:10.3390/ijms25105445)
Supplement: Supplementary file 1 [file ijms-25-05445-s001.zip › Supplemental material S5. Top10 DMPs.pdf]

Additional file S5. Top 10 differentially methylated CpGs with the highest methylation Beta difference in each comparison performed.

sEOAD vs. CTRL							
BRAIN				LCLs			
CpG	Gene	Beta difference	Adj.pval	CpG	Gene	Beta difference	Adj.pval
cg00892228	*TAS1R2*	0.4916	0.0008	cg02333792	*RASGRF1*	0.5822	0.0002
cg14114910	*MORN5*	0.4771	0.0000	cg01165781	*SYTL1*	-0.4866	0.0029
cg11973981	*ISPD*	0.4759	0.0450	cg19317532	*LRRC1*	-0.4764	0.0000
cg00348031	*NFATC1*	-0.4712	0.0120	cg26397736	*IL4I1; NUP62*	-0.4715	0.0001
cg12169700	*MAD1L1*	-0.4670	0.0205	cg15967058	*AKR1B1*	-0.4660	0.0001
cg16178271	*MAD1L1*	-0.4469	0.0184	cg10193422	*MAX*	0.4647	0.0000
cg17181941	*NCAPH*	-0.4435	0.0011	cg16797831	*KIAA1324; C1orf194*	0.4640	0.0000
cg22481673	*RD3*	0.4384	0.0011	cg11120684	*BACH2*	-0.4424	0.0000
cg14866535	*FAM65C*	0.4218	0.0021	cg25004725	*ANXA6*	0.4396	0.0007
cg11008123	*LOC283267*	0.4121	0.0032	cg24937675	*SGK1*	-0.4376	0.0051

PSEN1 vs. CTRL							
BRAIN				LCLs			
CpG	Gene	Beta difference	Adj.pval	CpG	Gene	Beta difference	Adj.pval
cg05611160	*LPPR1*	-0.5632	0.0090	cg03013609	*LGALS8*	-0.5929	0.0017
cg14417873	*VAV2*	-0.5237	0.0051	cg14252149	*LGALS8*	-0.5806	0.0022
cg06508795	*DDC*	0.4949	0.0133	cg09993319	*MGMT*	0.5719	0.0033
cg01462799	*UPF1*	0.4874	0.0004	cg26839010	*C1orf87*	0.5367	0.0000
cg04026937	*HLA-DRB1*	0.4807	0.0004	cg03544800	*DNTTIP2*	-0.5202	0.0025
cg17858192	*PROM1*	0.4648	0.0055	cg00350405	*IWS1*	0.4960	0.0003
cg01326874	*ZNF714*	-0.4542	0.0021	cg09114339	*PTPN1*	0.4603	0.0005
cg03013609	*LGALS8*	-0.4392	0.0020	cg17572255	*LIMA1*	-0.4466	0.0009
cg06174194	*PIK3CG*	0.4247	0.0008	cg04797323	*SOCS2*	-0.4411	0.0013
cg17487799	*CYP7B1*	-0.4233	0.0027	cg09829645	*DYSF*	0.4343	0.0056

PSEN1 vs. sEOAD							
BRAIN				LCLs			
CpG	Gene	Beta difference	Adj.pval	CpG	Gene	Beta difference	Adj.pval
cg14417873	*VAV2*	-0.5307	0.0017	cg11579905	*LRRIQ3; TNNI3K; FPGT*	0.5778	0.0000

cg00348031	*NFATC1*	0.5015	0.0401	cg27515399	*HERPUD1*	0.5288	0.0000
cg08238516	*NSF*	0.4564	0.0000	cg16885861	*CD81*	0.5043	0.0027
cg06961873	*TMEM57*	0.4534	0.0000	cg04588766	*SMARCC1*	0.4997	0.0001
cg01326874	*ZNF714*	-0.4494	0.0000	cg26851891	*MEF2C*	0.4932	0.0000
cg18443741	*PYROXD1*	0.4454	0.0061	cg13505479	*FAM60A*	0.4891	0.0034
cg17351927	*NOTCH4*	0.4337	0.0013	cg23599843	*JPH1*	-0.4842	0.0000
cg05867245	*ZBTB46*	0.4067	0.0249	cg14789659	*ARID5B*	0.4815	0.0001
cg21829038	*LRRK1*	0.4027	0.0000	cg09209679	*FAM19A5*	0.4791	0.0025
cg09352518	*ZNF714*	-0.3987	0.0000	cg08095377	*C2orf27A*	0.4747	0.0000

**sFTD-Tau vs. CTRL (Brain)**				**sFTD-TDP43 vs. CTRL (Brain)**			
**CpG**	**Gene**	**Beta difference**	**Adj.pval**	**CpG**	**Gene**	**Beta difference**	**Adj.pval**
cg15184869	*PEBP4*	-0.6193	0.0001	cg13569207	*ZNF862*	0.6267	0.0005
cg07505631	*CDCP1*	0.5261	0.0028	cg13913990	*PLEKHG4B*	-0.5341	0.0034
cg06508795	*DDC*	0.4930	0.0228	cg25285484	*ZNF597; NAA60*	0.4985	0.0012
cg05138546	*KRT36*	-0.4604	0.0136	cg08024264	*SCARNA16; C17orf86*	0.4966	0.0002
cg10240906	*BMP7*	0.4588	0.0413	cg17925226	*BMPR1B*	0.4702	0.0000
cg11229715	*CUL3*	0.4417	0.0126	cg18391209	*CAPN8*	0.4635	0.0000
cg00729708	*LASS3*	0.4374	0.0000	cg00729708	*LASS3*	0.4625	0.0000
cg17056069	*ASAP2*	-0.4348	0.0135	cg14651435	*DNAJB6*	0.4387	0.0207
cg11969330	*CYP4V2*	0.4223	0.0000	cg12657416	*FAM69B*	0.4256	0.0003
cg06091288	*SORBS1*	0.4180	0.0003	cg05305893	*FGF11; CHRNB1*	0.4243	0.0000

**sFTD-TDP43 vs. sFTD-Tau (Brain)**			
**CpG**	**Gene**	**Beta difference**	**Adj.pval**
cg10890644	*TUBAL3*	0.6517	0.0000
cg26983017	*HCK*	0.5955	0.0151
cg20381372	*LOC100134317*	0.5890	0.0000
cg09650803	*IL4R*	-0.5454	0.0002
cg18932722	*TMCC3*	-0.5441	0.0116
cg10006614	*C14orf139*	0.5313	0.0037
cg25285484	*ZNF597; NAA60*	0.4630	0.0404
cg08283200	*SOX5*	-0.4605	0.0000
cg01553548	*FRAS1*	0.4399	0.0203
cg27049594	*OR8A1*	0.4330	0.0132

MAPT vs. CTRL							
BRAIN				LCLs			
CpG	Gene	Beta difference	Adj.pval	CpG	Gene	Beta difference	Adj.pval
cg06409673	*FAM19A5*	-0.5927	0.0497	cg20592836	*TP53INP2*	0.6685	0.0010
cg03958058	*PPP2R2D*	0.5544	0.0005	cg16763089	*LOC149837*	0.5710	0.0018
cg11973981	*ISPD*	0.5498	0.0001	cg21778743	*FAM228A*	0.5533	0.0000
cg07505631	*CDCP1*	0.5431	0.0015	cg23713742	*SPAG4*	0.5497	0.0069
cg00588575	*SAPCD2*	0.5306	0.0000	cg26217827	*ITGA11*	-0.5471	0.0000
cg21211688	*ADAMTSL2*	-0.5306	0.0409	cg15768146	*ZFHX2; THTPA*	0.5467	0.0001
cg12657416	*FAM69B*	0.5167	0.0011	cg20295248	*LOC149837*	0.5423	0.0003
cg14866535	*FAM65C*	0.4837	0.0000	cg11897887	*CST9L*	-0.5357	0.0010
cg10482512	*CCR6*	0.4703	0.0012	cg19656689	*PTPRR*	0.5256	0.0001
cg15950547	*FAM107B*	0.4573	0.0000	cg19317715	*AOC2*	-0.5177	0.0010

GRN vs. CTRL							
BRAIN				LCLs			
CpG	Gene	Beta difference	Adj.pval	CpG	Gene	Beta difference	Adj.pval
cg03958058	*PPP2R2D*	0.6698	0.0000	cg08919780	*CDK13*	-0.6478	0.0000
cg10632770	*KIAA1199*	-0.5623	0.0189	cg00540295	*FAM69B*	0.5596	0.0085
cg04132418	*LOC285733*	0.5571	0.0109	cg18816397	*HLA-DRB5*	-0.5442	0.0000
cg00892228	*TAS1R2*	0.5513	0.0067	cg14252149	*LGALS8*	-0.5372	0.0029
cg12206353	*ARHGEF28*	-0.5258	0.0001	cg03013609	*LGALS8*	-0.5247	0.0067
cg14168080	*PTPRN2*	0.4933	0.0000	cg01002086	*FSTL4*	0.4995	0.0001
cg18624102	*FBXO27*	0.4927	0.0447	cg25004725	*ANXA6*	0.4966	0.0010
cg04453550	*TNFRSF17*	-0.4832	0.0001	cg11377625	*KCTD1*	-0.4862	0.0000
cg06508795	*DDC*	0.4811	0.0186	cg13616508	*YWHAG*	0.4721	0.0000
cg22984586	*CCR5*	0.4670	0.0047	cg26651280	*GALNS*	-0.4715	0.0082

C9orf72 vs. CTRL (Brain)			
CpG	Gene	Beta difference	Adj.pval
cg13569207	*ZNF862*	0.6605	0.0007
cg21211688	*ADAMTSL2*	-0.6461	0.0000
cg11008123	*LOC283267*	0.5847	0.0087
cg04026937	*HLA-DRB1*	0.5440	0.0010
cg04683516	*AKR1C2*	-0.5356	0.0216

cg03958058	*PPP2R2D*	0.5128	0.0092
cg21388339	*TP73*	-0.4735	0.0006
cg14889167	*FSTL4*	0.4728	0.0458
cg20976286	*OCA2*	0.4508	0.0055
cg06174194	*PIK3CG*	0.4376	0.0002

Sporadic FTD and *C9orf72* patients had not LCLs samples available. In comparisons between patients and controls, hyper or hypomethylation refers to the patients' group. Between two groups of patients, it refers to the second group of the comparison. Abbreviations: CTRL, healthy controls; sEOAD, sporadic early-onset Alzheimer's disease; PSEN1, autosomal dominant Alzheimer's disease caused by mutation in *PSEN1*; MAPT, GRN, C9orf72, familial frontotemporal dementia caused by mutation in *MAPT*, *GRN* or *C9orf72*; sFTD-Tau, sporadic frontotemporal dementia with tau deposits; sFTD-TDP43, sporadic frontotemporal dementia with TDP43 deposits; LCLs, lymphoblastoid cell lines.
