# Supplementary material for: Genome-Wide DNA Methylation in Early-Onset-Dementia Patients Brain Tissue and Lymphoblastoid Cell Lines
Source: Int J Mol Sci. 2024 May 16;25(10):5445. doi: 10.3390/ijms25105445 (PMC11121630; doi:10.3390/ijms25105445)
Supplement: Supplementary file 1 [file ijms-25-05445-s001.zip › Supplemental material S6. Pathways_GO.pdf]

Additional file S6. Biological processes (BPs) obtained in AD and FTD comparisons applying GO database.

sEOAD vs. CTRL					
**BRAIN**			**LCLs**		
**Biological process**	**Enrichment FDR**	**Root node**	**Biological process**	**Enrichment FDR**	**Root node**
Extracellular matrix assembly	0.026	Cellular component organization	Activation of protein kinase activity	0.021	Metabolic process
Cell adhesion	0.026	//	Positive regulation of extrinsic apoptotic signaling pathway via death domain receptors	0.030	Regulation of biological process
Biological adhesion	0.026	//	Locomotory behavior	0.030	Behavior
			Positive regulation of signal transduction	0.030	Regulation of biological process
			Astral microtubule organization	0.030	Cellular process
			Ion transmembrane transport	0.030	Transport
			Regulation of protein kinase activity	0.030	Regulation of biological process
			Genetic imprinting	0.030	Regulation of biological process
			Intracellular signal transduction	0.032	//
			Response to endogenous stimulus	0.041	Response to stimulus
			Regulation of transferase activity	0.041	Biological regulation
			Calcium ion transmembrane transport	0.041	Transport
			Inorganic ion transmembrane transport	0.050	Transport

PSEN1 vs. CTRL					
**BRAIN**			**LCLs**		
**Biological process**	**Enrichment FDR**	**Root node**	**Biological process**	**Enrichment FDR**	**Root node**
Phospholipid transport	0.002	Macromolecule localization	Homophilic cell adhesion via plasma membrane adhesion molecules	0.000	Cell adhesion

Sterol metabolic process	0.002	Metabolism
Phospholipid translocation	0.005	Macromolecule localization
Lipid translocation	0.005	Macromolecule localization
Steroid metabolic process	0.005	Metabolism
Organophosphate ester transport	0.008	Macromolecule localization
Organic hydroxy compound metabolic process	0.009	Metabolism
Cholesterol metabolic process	0.022	Metabolism
Regulation of membrane lipid distribution	0.024	Cellular component organization
Secondary alcohol metabolic process	0.024	Metabolism
Axonogenesis	0.029	Cell development
Anterior commissure morphogenesis	0.031	Cell development
Regulation of ATP metabolic process	0.034	Metabolism
Central nervous system projection neuron axonogenesis	0.034	Cell development
Regulation of modification of postsynaptic actin cytoskeleton	0.037	Cellular component organization
Lipid metabolic process	0.037	Metabolism
Axon development	0.037	Cell development
Neuron projection guidance	0.043	Cell development
Regulation of modification of postsynaptic structure	0.043	Cellular component organization
Regulation of purine nucleotide metabolic process	0.043	Metabolism
Alcohol metabolic process	0.043	Metabolism
Organic hydroxy compound biosynthetic process	0.043	Metabolism

Cell-cell adhesion via plasma-membrane adhesion molecules	0.000	Cell adhesion
Cell-cell adhesion	0.011	Cell adhesion
Cell adhesion	0.031	//
Plasma membrane repair	0.031	Cellular process
Biological adhesion	0.031	Cell adhesion
Negative regulation of dendritic spine development	0.031	Developmental process
Cholesterol metabolic process	0.038	Metabolic process
Secondary alcohol metabolic process	0.040	Metabolic process
Plasma membrane organization	0.040	Cellular process
Import into cell	0.049	Transport

Regulation of glycolytic process	0.043	Metabolism			
Regulation of nucleotide metabolic process	0.043	Metabolism			
Cell morphogenesis involved in differentiation	0.043	Developmental process			
Axon guidance	0.043	Cell development			
Response to external stimulus	0.043	Response to stimulus			
Organic acid biosynthetic process	0.043	Metabolism			
Central nervous system neuron axonogenesis	0.043	Cell development			
Regulation of nucleotide catabolic process	0.043	Metabolism			
**PSEN1 vs. sEOAD**					
**BRAIN**			**LCLs**		
**Biological process**	**Enrichment FDR**	**Root node**	**Biological process**	**Enrichment FDR**	**Root node**
Endothelial cell morphogenesis	0.005	Developmental process	Phosphorus metabolic process	0.001	Metabolic process
Cellular component morphogenesis	0.022	Developmental process	Phosphate-containing compound metabolic process	0.001	Metabolic process
Epithelial cell morphogenesis	0.028	Developmental process	Negative regulation of phosphorus metabolic process	0.001	Regulation of biological process
Positive regulation of lipid kinase activity	0.029	Regulation of metabolic process	Dephosphorylation	0.001	Metabolic process
Anatomical structure formation involved in morphogenesis	0.038	Developmental process	Negative regulation of protein phosphorylation	0.001	Regulation of biological process
Positive regulation of phospholipid metabolic process	0.044	Regulation of metabolic process	Regulation of signaling	0.001	Regulation of biological process
Negative regulation of dendritic cell differentiation	0.050	Cell differentiation	Negative regulation of protein modification process	0.001	Regulation of biological process
Cell morphogenesis	0.050	Developmental process	Negative regulation of phosphate metabolic process	0.001	Regulation of biological process
Anatomical structure morphogenesis	0.050	Developmental process	Regulation of cell communication	0.001	Regulation of biological process
Positive regulation of cell morphogenesis involved in differentiation	0.050	Developmental process	Negative regulation of cellular metabolic process	0.001	Regulation of biological process
Endothelial cell development	0.050	Developmental process	Negative regulation of cellular protein metabolic process	0.001	Regulation of biological process

Tube development	0.050	Developmental process
Regulation of lipid kinase activity	0.050	Regulation of metabolic process

Negative regulation of protein serine/threonine kinase activity	0.001	Regulation of biological process
Negative regulation of nitrogen compound metabolic process	0.001	Regulation of biological process
Negative regulation of intracellular signal transduction	0.001	Regulation of biological process
Negative regulation of protein metabolic process	0.001	Regulation of biological process
Negative regulation of phosphorylation	0.002	Regulation of biological process
Regulation of signal transduction	0.002	Regulation of biological process
Anatomical structure morphogenesis	0.003	Developmental process
Negative regulation of protein kinase activity	0.004	Regulation of biological process
Regulation of catalytic activity	0.004	Regulation of biological process
Protein dephosphorylation	0.004	Metabolic process
Negative regulation of macromolecule metabolic process	0.004	Regulation of biological process
Negative regulation of MAP kinase activity	0.004	Regulation of biological process
Negative regulation of MAPK cascade	0.004	Regulation of biological process
Nervous system development	0.005	Developmental process
Negative regulation of metabolic process	0.005	Regulation of biological process
Negative regulation of cell communication	0.006	Regulation of biological process
Negative regulation of signaling	0.006	Regulation of biological process
Osteoclast development	0.006	Developmental process
Negative regulation of kinase activity	0.006	Regulation of biological process

sFTD-Tau vs. CTRL			sFTD-TDP43 vs. CTRL		
**Biological process**	**Enrichment FDR**	**Root node**	**Biological process**	**Enrichment FDR**	**Root node**
Neuron projection guidance	0.003	Developmental process	Cell adhesion	0.000	Biological adhesion
Nervous system development	0.003	Developmental process	Biological adhesion	0.000	//
Axon guidance	0.003	Developmental process	Homophilic cell adhesion via plasma membrane adhesion molecules	0.000	Biological adhesion
Cell morphogenesis involved in differentiation	0.004	Developmental process	Cell-cell adhesion via plasma-membrane adhesion molecules	0.000	Biological adhesion
Cell development	0.004	Developmental process	Cell-cell adhesion	0.000	Biological adhesion
Cell morphogenesis involved in neuron differentiation	0.004	Developmental process	Cell morphogenesis involved in neuron differentiation	0.000	Developmental process
Axonogenesis	0.004	Developmental process	Cell morphogenesis involved in differentiation	0.000	Developmental process
Central nervous system development	0.005	Developmental process	Neuron projection development	0.000	Developmental process
Neuron differentiation	0.005	Developmental process	Cell morphogenesis	0.000	Developmental process
Neuron projection morphogenesis	0.008	Developmental process	Neuron development	0.000	Developmental process
Axon development	0.008	Developmental process	Dendrite development	0.000	Developmental process
Plasma membrane bounded cell projection morphogenesis	0.009	Developmental process	Cellular component morphogenesis	0.000	Developmental process
Cell projection morphogenesis	0.009	Developmental process	Neuron projection morphogenesis	0.000	Developmental process
Cell part morphogenesis	0.012	Developmental process	Anatomical structure morphogenesis	0.000	Developmental process
Neuron development	0.012	Developmental process	Plasma membrane bounded cell projection morphogenesis	0.000	Developmental process
Generation of neurons	0.012	Developmental process	Cell projection morphogenesis	0.000	Developmental process
Head development	0.014	Developmental process	Axonogenesis	0.000	Developmental process
Neuron migration	0.014	Developmental process	Cell part morphogenesis	0.000	Developmental process
Brain development	0.015	Developmental process	Cell development	0.000	Developmental process

Movement of cell or subcellular component	0.015	Cellular process	Movement of cell or subcellular component	0.000	Cellular process
Neurogenesis	0.015	Developmental process	Generation of neurons	0.000	Developmental process
Cell differentiation	0.015	Developmental process	Neurogenesis	0.000	Developmental process
Response to transforming growth factor beta	0.016	Response to stimulus	Neuron differentiation	0.000	Developmental process
Anatomical structure morphogenesis	0.020	Developmental process	Axon development	0.000	Developmental process
Cell morphogenesis	0.028	Developmental process	Cell projection organization	0.000	Cellular process
Neuron projection development	0.028	Developmental process	Synapse organization	0.000	Cellular process
Forebrain development	0.029	Developmental process	Nervous system development	0.000	Developmental process
Positive regulation of caveolin-mediated endocytosis	0.030	Regulation of biological process	Plasma membrane bounded cell projection organization	0.000	Cellular process
Telencephalon development	0.032	Developmental process	Cell differentiation	0.000	Developmental process
Cellular localization	0.034	Localization	Regulation of cell differentiation	0.000	Developmental process

sFTD-TDP43 vs. sFTD-Tau		
**Biological process**	**Enrichment FDR**	**Root node**
Response to phorbol 13-acetate 12-myristate	0.031	Response to stimulus
Cellular response to phorbol 13-acetate 12-myristate	0.031	Response to stimulus
Response to biotic stimulus	0.031	Response to stimulus

MAPT vs. CTRL					
BRAIN			LCLs		
**Biological process**	**Enrichment FDR**	**Root node**	**Biological process**	**Enrichment FDR**	**Root node**
Cell morphogenesis involved in differentiation	0.001	Developmental process	Nervous system development	0.000	Developmental process
Movement of cell or subcellular component	0.001	Cellular process	Cell development	0.000	Developmental process
Axonogenesis	0.001	Developmental process	Anatomical structure morphogenesis	0.000	Developmental process
Cell development	0.001	Developmental process	Neurogenesis	0.000	Developmental process
Axon development	0.001	Developmental process	Generation of neurons	0.000	Developmental process
Plasma membrane bounded cell projection morphogenesis	0.002	Developmental process	Neuron differentiation	0.000	Developmental process
Negative regulation of cellular protein localization	0.002	Biological regulation	Cellular component morphogenesis	0.000	Developmental process
Cell morphogenesis	0.002	Developmental process	Movement of cell or subcellular component	0.000	Cellular process
Neuron differentiation	0.002	Developmental process	Locomotion	0.000	Locomotion
Cellular component morphogenesis	0.002	Developmental process	Cellular developmental process	0.000	Developmental process
Neuron development	0.002	Developmental process	Neuron development	0.000	Developmental process
Cell morphogenesis involved in neuron differentiation	0.002	Developmental process	Regulation of localization	0.000	Regulation of biological process
Neuron projection morphogenesis	0.002	Developmental process	Neuron projection development	0.000	Developmental process
Cell projection morphogenesis	0.002	Developmental process	Cell differentiation	0.000	Developmental process
Anatomical structure morphogenesis	0.002	Developmental process	Cell morphogenesis	0.000	Developmental process
Neuron projection development	0.002	Developmental process	Plasma membrane bounded cell projection organization	0.000	Cellular process
Chondrocyte differentiation	0.002	Developmental process	Cell morphogenesis involved in neuron differentiation	0.000	Developmental process

Cell part morphogenesis	0.002	Developmental process	Cell motility	0.000	Locomotion
Plasma membrane bounded cell projection organization	0.006	Cellular process	Localization of cell	0.000	Localization
Nervous system development	0.008	Developmental process	Cell-cell signaling	0.000	Signaling
Negative regulation of protein localization to membrane	0.009	Localization	Cell projection organization	0.000	Cellular process
Cell projection organization	0.009	Cellular process	Actin filament-based process	0.000	Cellular process
Regulation of GTPase activity	0.010	Biological regulation	Plasma membrane bounded cell projection morphogenesis	0.000	Developmental process
Regulation of hydrolase activity	0.010	Biological regulation	Cell part morphogenesis	0.000	Developmental process
Generation of neurons	0.013	Developmental process	Cell projection morphogenesis	0.000	Developmental process
Cell adhesion	0.014	Biological adhesion	Cell migration	0.000	Locomotion
Central nervous system neuron axonogenesis	0.014	Developmental process	Cytoskeleton organization	0.000	Cellular process
Cell differentiation	0.014	Developmental process	Cell-cell adhesion via plasma-membrane adhesion molecules	0.000	Cell adhesion
Regulation of catalytic activity	0.014	Biological regulation	Regulation of biological quality	0.000	Regulation of biological process
Regulation of membrane repolarization	0.014	Biological regulation	Cell morphogenesis involved in differentiation	0.000	Developmental process

**GRN vs. CTRL**					
**BRAIN**			**LCLs**		
**Biological process**	**Enrichment FDR**	**Root node**	**Biological process**	**Enrichment FDR**	**Root node**
Response to organic substance	0.000	Response to stimulus	Negative regulation of multicellular organism growth	0.005	Regulation of biological process
Cell morphogenesis	0.000	Developmental process	Negative regulation of growth	0.005	Regulation of biological process
Movement of cell or subcellular component	0.000	Cellular process	Response to hydroperoxide	0.014	Response to stimulus
Cell adhesion	0.000	Biological adhesion	Negative regulation of developmental growth	0.014	Regulation of biological process
Biological adhesion	0.000	//			

Cellular component morphogenesis	0.000	Response to stimulus
Locomotion	0.000	//
Cell motility	0.000	Locomotion
Localization of cell	0.000	Localization
Cell migration	0.000	Locomotion
Cellular response to organic substance	0.000	Response to stimulus
Blood vessel development	0.000	Developmental process
Cell proliferation	0.000	Developmental process
Vasculature development	0.000	Developmental process
Extracellular matrix organization	0.000	Cellular process
Cell-substrate adhesion	0.000	Biological adhesion
Cardiovascular system development	0.000	Developmental process
Nervous system development	0.000	Developmental process
Homophilic cell adhesion via plasma membrane adhesion molecules	0.001	Biological adhesion
Circulatory system development	0.001	Developmental process
Extracellular structure organization	0.001	Cellular process
Regulation of cell proliferation	0.001	Biological regulation
Anatomical structure morphogenesis	0.001	Developmental process
Response to oxygen-containing compound	0.001	Response to stimulus
Anatomical structure formation involved in morphogenesis	0.001	Developmental process
Cellular response to chemical stimulus	0.001	Response to stimulus
Cell morphogenesis involved in differentiation	0.001	Developmental process
Regulation of biological quality	0.002	Biological regulation
Angiogenesis	0.002	Developmental

		process
Enzyme linked receptor protein signaling pathway	0.002	Signaling

For each BPs, the FDR and the root node in which the BPs belongs is shown. LCLs samples of C9orf72 group and sporadic FTD were not available.C9orf72 did not retrieve any BPs. Filters applied: adjusted-p value <0.05. Abbreviations: CTRL, healthy controls; sEOAD, sporadic early-onset Alzheimer's disease; PSEN1, autosomal dominant Alzheimer's disease caused by mutation in *PSEN1*; MAPT, GRN, C9orf72, familial frontotemporal dementia caused by mutation in *MAPT*, *GRN* or *C9orf72*; sFTD-Tau, sporadic frontotemporal dementia with tau deposits; sFTD-TDP43, sporadic frontotemporal dementia with TDP43 deposits; LCLs, lymphoblastoid cell lines.
