# Supplementary material for: Genome-Wide DNA Methylation in Early-Onset-Dementia Patients Brain Tissue and Lymphoblastoid Cell Lines
Source: Int J Mol Sci. 2024 May 16;25(10):5445. doi: 10.3390/ijms25105445 (PMC11121630; doi:10.3390/ijms25105445)
Supplement: Supplementary file 1 [file ijms-25-05445-s001.zip › Supplemental material S7. Pathways_Reactome.pdf]

Additional file S7. Pathways found in AD and FTD comparisons applying Reactome database.

sEOAD vs. CTRL					
BRAIN			LCLs		
Biological process	FDR	Root node	Biological process	p-value	Root node
Rhesus blood group biosynthesis	0.016	Metabolism	Transcriptional Regulation by MECP2	0.003	Gene expression
Cytokine Signaling in Immune system	0.017	Immune system	Activation of anterior HOX genes in hindbrain development during early embryogenesis	0.006	Developmental biology
			Activation of HOX genes during differentiation	0.006	Developmental biology
			Keratan sulfate degradation	0.008	Metabolism
			Transport of gamma-carboxylated protein precursors from the endoplasmic reticulum to the Golgi apparatus	0.012	Metabolism
			CD28 dependent PI3K/Akt signaling	0.012	Immune system
			Ras activation upon Ca2+ influx through NMDA receptor	0.014	Neuronal system
			Ca2+ activated K+ channels	0.015	Neuronal system
			MECP2 regulates transcription factors	0.015	Gene expression
			Removal of aminoterminal propeptides from gamma-carboxylated proteins	0.015	Metabolism
			Unblocking of NMDA receptors, glutamate binding and activation	0.015	Neuronal system
			Long-term potentiation	0.020	Neuronal system
			Apoptosis induced DNA fragmentation	0.024	Programmed cell death
			Synthesis of bile acids and bile salts via 27-hydroxycholesterol	0.025	Metabolism
			PECAM1 interactions	0.028	Hemostasis
			Activation of the pre-replicative complex	0.029	DNA replication
			DNA replication initiation	0.035	DNA replication

			Gamma-carboxylation, transport, and amino-terminal cleavage of proteins	0.035	Metabolism
			CD28 co-stimulation	0.035	Immune system
			CREB1 phosphorylation through NMDA receptor-mediated activation of RAS signaling	0.035	Neuronal system
			Synthesis of IP3 and IP4 in the cytosol	0.035	Metabolism
			SUMOylation of intracellular receptors	0.038	Metabolism
			Reversible hydration of carbon dioxide	0.039	Metabolism
			RUNX1 and FOXP3 control the development of regulatory T lymphocytes (Tregs)	0.039	Gene expression
			cGMP effects	0.043	Hemostasis
			HS-GAG degradation	0.048	Metabolism
			Interleukin-2 signaling	0.048	Immune system
**PSEN1 vs. CTRL**					
**BRAIN**			**LCLs**		
**Biological process**	**p-value**	**Root node**	**Biological process**	**p-value**	**Root node**
GRB7 events in ERBB2 signaling	0.003	Signal transduction	DARPP-32 events	0.003	Signal transduction
FOXO-mediated transcription of oxidative stress, metabolic and neuronal genes	0.003	Gene expression	Calcineurin activates NFAT	0.006	Immune system
NR1H2 and NR1H3-mediated signaling	0.004	Signal transduction	NR1H2 & NR1H3 regulate gene expression linked to lipogenesis	0.009	Signal transduction
O-linked glycosylation	0.006	Metabolism	CLEC7A (Dectin-1) induces NFAT activation	0.010	Immune system
RUNX3 Regulates Immune Response and Cell Migration	0.007	Gene expression	MGMT-mediated DNA damage reversal	0.017	DNA repair
Semaphorin interactions	0.012	Developmental biology	RORA activates gene expression	0.019	Circadian clock
O-linked glycosylation of mucins	0.013	Metabolism	Defective TPMT causes Thiopurine S-methyltransferase deficiency (TPMT deficiency)	0.025	Disease

LGI-ADAM interactions	0.013	Developmental biology	Defective SLC12A6 causes agenesis of the corpus callosum, with peripheral neuropathy (ACCPN)	0.025	Disease
Downregulation of ERBB2:ERBB3 signaling	0.017	Signal transduction	Growth hormone receptor signaling	0.026	Immune system
NR1H2 & NR1H3 regulate gene expression linked to lipogenesis	0.019	Signal transduction	Activated NOTCH1 Transmits Signal to the Nucleus	0.031	Signal transduction
CRMPs in Sema3A signaling	0.021	Developmental biology	MECP2 regulates neuronal receptors and channels	0.031	Gene expression
ERBB2 Activates PTK6 Signaling	0.021	Signal transduction	Glycogen storage disease type 0 (muscle GYS1)	0.033	Disease
ERBB2 Regulates Cell Motility	0.023	Signal transduction	Glycogen storage disease type XV (GYG1)	0.033	Disease
Other semaphorin interactions	0.023	Developmental biology	Glycogen storage disease type II (GAA)	0.033	Disease
PI3K events in ERBB2 signaling	0.030	Signal transduction	Interleukin-7 signaling	0.038	Immune system
PI and PC transport between ER and Golgi membranes	0.036	Metabolism	Integrin cell surface interactions	0.039	Extracellular matrix organization
Defective ALG1 causes ALG1-CDG (CDG-1k)	0.036	Disease	The NLRP1 inflammasome	0.042	Immune system
EPH-Ephrin signaling	0.036	Developmental biology	GABA synthesis	0.050	Neuronal system
NRAGE signals death through JNK	0.043	Signal transduction	MECP2 regulates transcription of genes involved in GABA signaling	0.050	Gene expression
Termination of O-glycan biosynthesis	0.047	Metabolism			
Notch-HLH transcription pathway	0.047	Gene expression			
FOXO-mediated transcription	0.047	Gene expression			
NR1H3 & NR1H2 regulate gene expression linked to cholesterol transport and efflux	0.048	Signal transduction			
**PSEN1 vs. sEOAD**					
**BRAIN**			**LCLs**		
**Biological process**	**p-value**	**Root node**	**Biological process**	**p-value**	**Root node**
GABA synthesis, release, reuptake and degradation	0.001	Neuronal system	MECP2 regulates transcription factors	0.008	Gene expression

Neurotransmitter release cycle	0.002	Neuronal system	Glutathione conjugation	0.013	Metabolism
Transmission across Chemical Synapses	0.003	Neuronal system	MGMT-mediated DNA damage reversal	0.026	DNA repair
CD28 dependent PI3K/Akt signaling	0.009	Immune system	Aflatoxin activation and detoxification	0.028	Metabolism
Integrin cell surface interactions	0.012	Extracellular matrix organization	Defective SLC6A3 causes Parkinsonism-dystonia infantile (PKDYS)	0.039	Disease
Laminin interactions	0.012	Extracellular matrix organization	Defective SLC6A3 causes Parkinsonism-dystonia infantile (PKDYS)	0.039	Disease
Pre-NOTCH Transcription and Translation	0.012	Signal transduction	Defective GGT1 causes Glutathionuria (GLUTH)	0.039	Disease
Glutamate Neurotransmitter Release Cycle	0.013	Neuronal system	Defective SLC9A9 causes autism 16 (AUTS16)	0.039	Disease
Costimulation by the CD28 family	0.017	Immune system	XAV939 stabilizes AXIN	0.039	Disease
Neuronal System	0.018	//	Digestion of dietary lipid	0.041	Digestion and absorption
CD28 co-stimulation	0.019	Immune system	Synthesis of Ketone Bodies	0.041	Metabolism
GPVI-mediated activation cascade	0.023	Hemostasis	RORA activates gene expression	0.044	Circadian clock
Pre-NOTCH Expression and Processing	0.023	Signal transduction	Negative regulation of MET activity	0.044	Signal transduction
VEGFR2 mediated vascular permeability	0.024	Signal transduction	Glutathione synthesis and recycling	0.047	Metabolism
Collagen chain trimerization	0.024	Extracellular matrix organization	Amino acids regulate mTORC1	0.048	Cellular response to external stimulus
MET interacts with TNS proteins	0.026	Signal transduction			
Defective LFNG causes SCDO3	0.032	Disease			
Platelet Aggregation (Plug Formation)	0.033	Hemostasis			
VEGFA-VEGFR2 Pathway	0.035	Signal transduction			
Activation of AKT2	0.037	Signal transduction			
Toxicity of botulinum toxin type G (botG)	0.037	Disease			
Pyruvate metabolism	0.038	Metabolism			
Signaling by VEGF	0.043	Signal transduction			
Interferon gamma signaling	0.049	Immune system			

sFTD-Tau vs. CTRL		
**Biological process**	**p-value**	**Root node**
RUNX3 regulates YAP1-mediated transcription	0.001	Gene expression
SHC1 events in ERBB4 signaling	0.001	Signal transduction
GRB2 events in ERBB2 signaling	0.001	Signal transduction
SHC1 events in ERBB2 signaling	0.001	Signal transduction
Binding of TCF/LEF:CTNNB1 to target gene promoters	0.002	Signal transduction
Downregulation of ERBB4 signaling	0.002	Signal transduction
ROBO receptors bind AKAP5	0.002	Developmental biology
Nitric oxide stimulates guanylate cyclase	0.003	Hemostasis
Nuclear signaling by ERBB4	0.005	Signal transduction
PI3K events in ERBB4 signaling	0.005	Signal transduction
Signaling by Receptor Tyrosine Kinases	0.005	Signal transduction
DAG and IP3 signaling	0.008	Signal transduction
Phosphate bond hydrolysis by NUDT proteins	0.008	Metabolism
HDL assembly	0.008	Transport of small molecules
GRB7 events in ERBB2 signaling	0.008	Signal transduction
Signaling by ERBB2 TMD/JMD mutants	0.009	Disease
Signaling by ERBB2	0.009	Signal transduction
Downregulation of ERBB2 signaling	0.010	Signal transduction

sFTD-TDP43 vs. CTRL		
**Biological process**	**FDR**	**Root node**
Downstream TCR signaling	0.000	Immune system
TCR signaling	0.000	Immune system

sFTD-TDP43 vs. sFTD-Tau		
**Biological process**	**p-value**	**Root node**
Diseases of hemostasis	0.004	Disease
Defects of contact activation system (CAS) and kallikrein/kinin system (KKS)	0.004	Disease
Crosslinking of collagen fibrils	0.005	Extracellular matrix organization
Post-chaperonin tubulin folding pathway	0.005	Metabolism
Intrinsic Pathway of Fibrin Clot Formation	0.009	Hemostasis
Defective SERPING1 causes hereditary angioedema	0.013	Disease
Extracellular matrix organization	0.013	Extracellular matrix organization
Elastic fibre formation	0.016	Extracellular matrix organization
Defective factor XII causes hereditary angioedema	0.017	Disease
RHOBTB1 GTPase cycle	0.021	Signal transduction
NGF processing	0.021	Signal transduction
Expression and Processing of Neurotrophins	0.021	Signal transduction
Degradation of the extracellular matrix	0.025	Extracellular matrix organization
RHOBTB2 GTPase cycle	0.025	Signal transduction

			Formation of Fibrin Clot (Clotting Cascade)	0.028	Hemostasis
			Defective F9 activation	0.029	Disease
			COPI-independent Golgi-to-ER retrograde traffic	0.029	Vesicle-mediated transport
			Assembly of collagen fibrils and other multimeric structures	0.033	Extracellular matrix organization
			Nef and signal transduction	0.037	Disease
			Diseases associated with O-glycosylation of proteins	0.043	Disease
**MAPT vs. CTRL**					
**BRAIN**			**LCLs**		
**Biological process**	**p-value**	**Root node**	**Biological process**	**FDR**	**Root node**
RUNX3 regulates CDKN1A transcription	0.002	Gene expression	Nuclear Receptor transcription pathway	0.000	Gene expression
Opioid Signaling	0.007	Signal transduction			
O-glycosylation of TSR domain-containing proteins	0.008	Metabolism			
FOXO-mediated transcription of cell cycle genes	0.009	Gene expression			
**GRN vs. CTRL**					
**BRAIN**			**LCLs**		
**Biological process**	**FDR**	**Root node**	**Biological process**	**p-value**	**Root node**
Interferon gamma signaling	0.000	Immune system	MECP2 regulates transcription factors	0.003	Gene expression
			Adaptive Immune System	0.008	Immune system
			Defective F8 binding to von Willebrand factor	0.015	Disease
			Vitamin C (ascorbate) metabolism	0.017	Metabolism
			Growth hormone receptor signaling	0.021	Immune system

C9orf72 vs.CTRL		
**Biological process**	**FDR**	**Root node**
Collagen chain trimerization	0.000	Extracellular matrix organization

MPS IV - Morquio syndrome A	0.022	Disease
Glycogen storage disease type II (GAA)	0.030	Disease
Defective F8 cleavage by thrombin	0.030	Disease
Interleukin-7 signaling	0.031	Immune system
Maturation of spike protein	0.039	Disease

For each pathway, the FDR (or the p-value in case the FDR significance was not reached) and the root node in which the pathway belongs is shown. LCLs samples of C9orf72 group and sporadic FTD were not available. Filters applied: adjusted-p val <0.05. Abbreviations: CTRL, healthy controls; sEOAD, sporadic early-onset Alzheimer's disease; PSEN1, autosomal dominant Alzheimer's disease caused by mutation in *PSEN1*; MAPT, GRN, C9orf72, familial frontotemporal dementia caused by mutation in *MAPT*, *GRN* or *C9orf72*; sFTD-Tau, sporadic frontotemporal dementia with tau deposits; sFTD-TDP43, sporadic frontotemporal dementia with TDP43 deposits; LCLs, lymphoblastoid cell lines.
