# Supplementary material for: Genome-Wide DNA Methylation in Early-Onset-Dementia Patients Brain Tissue and Lymphoblastoid Cell Lines
Source: Int J Mol Sci. 2024 May 16;25(10):5445. doi: 10.3390/ijms25105445 (PMC11121630; doi:10.3390/ijms25105445)
Supplement: Supplementary file 1 [file ijms-25-05445-s001.zip › Supplemental material S8. Heatmaps_FTD.pdf]

Additional file S8. Heatmaps with the top 10 differentially methylated positions (DMPs) in some FTD comparisons performed in brain samples.

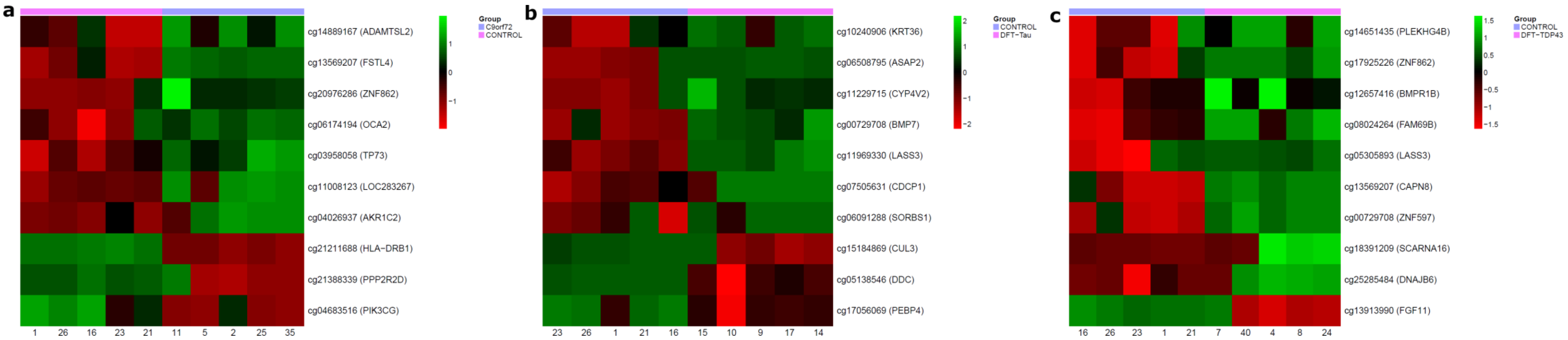

Each CpG obtained has the associated gene beside. (**a**) Brain C9orf72 vs. CTRL; (**b**) Brain sFTD-Tau vs. CTRL; (**c**) Brain sFTD-TDP43 vs. CTRL. Abbreviations: C9orf72, familial frontotemporal dementia caused by mutation in *C9orf72* gene; sFTD-Tau, sporadic frontotemporal dementia with tau deposits; sFTD-TDP43, sporadic frontotemporal dementia with TDP43 deposits; LCLs, lymphoblastoid cell lines.
