# Supplementary material for: Genome-Wide DNA Methylation in Early-Onset-Dementia Patients Brain Tissue and Lymphoblastoid Cell Lines
Source: Int J Mol Sci. 2024 May 16;25(10):5445. doi: 10.3390/ijms25105445 (PMC11121630; doi:10.3390/ijms25105445)
Supplement: Supplementary file 1 [file ijms-25-05445-s001.zip › Supplemental material S9. Common CpGs_FTD.pdf]

Additional file S9. Common differentially methylated CpGs (DMPs) found between FTD comparisons in brain.

Genetic Patients vs. Control							
		MAPT vs. CTRL		GRN vs. CTRL		C9orf72 vs. CTRL	
**CpG**	**Gene**	**Beta difference**	**Adj.pval**	**Beta difference**	**Adj.pval**	**Beta difference**	**Adj.pval**
cg17925226	*BMPR1B*	0.4267	0.0000	0.4659	0.0000	0.3109	0.0396
cg15829774	*BTBD8*	0.2624	0.0015	0.2631	0.0106	0.2514	0.0318
cg15950547	*FAM107B*	0.4573	0.0000	0.3335	0.0143	0.2913	0.0060
cg07902519	*MYBPC1*	0.3346	0.0108	0.3151	0.0164	0.3598	0.0048
cg06354054	*DHX37*	0.2989	0.0261	0.3303	0.0009	0.4019	0.0000
cg14168080	*PTPRN2*	0.3123	0.0380	0.4933	0.0000	0.2920	0.0263
cg13521643	*GNG10; DNAJC25-GNG10*	0.3253	0.0335	0.3193	0.0172	0.3306	0.0352
cg03958058	*PPP2R2D*	0.5544	0.0005	0.6698	0.0000	0.5128	0.0092
cg06174194	*PIK3CG*	0.2929	0.0030	0.3870	0.0001	0.4376	0.0002
cg15292356	*FOXK2*	0.3618	0.0191	0.3600	0.0006	0.3705	0.0074
cg00571519	*SRPK2*	0.3204	0.0483	0.4430	0.0020	0.3718	0.0391
cg17531142	*PVT1*	0.2611	0.0073	0.2648	0.0026	0.2675	0.0080

Sporadic Patients vs. Control					
		sFTD-Tau vs. CTRL		sFTD-TDP43 vs. CTRL	
**CpG**	**Gene**	**Beta difference**	**Adj.pval**	**Beta difference**	**Adj.pval**
cg17925226	*BMPR1B*	0.3718	0.0007	0.4702	0.0000
cg15829774	*BTBD8*	0.2576	0.0039	0.2643	0.0023
cg07902519	*MYBPC1*	0.2503	0.0048	0.3108	0.0025
cg00892228	*TAS1R2*	0.4058	0.0271	0.3074	0.0452
cg20428144	*APOLD1*	0.2882	0.0000	0.2755	0.0000
cg26178311	*DGCR14; TSSK2*	0.2821	0.0202	0.2971	0.0219
cg15411736	*CLECL1*	0.2723	0.0000	0.2688	0.0000
cg04242210	*DCLK1*	0.2926	0.0013	0.2746	0.0029
cg19393008	*KRT82*	0.2728	0.0379	0.2771	0.0075
cg27028557	*TPCN1*	0.2533	0.0095	0.2686	0.0000
cg04103432	*COL3A1; MIR1245A*	0.2574	0.0017	0.3027	0.0000
cg10737404	*USP13*	0.3940	0.0000	0.3676	0.0000
cg15846907	*PRKCA*	0.2678	0.0002	0.2808	0.0000
cg16645815	*INPP5A*	0.2608	0.0396	0.2860	0.0234
cg11969330	*CYP4V2*	0.4223	0.0000	0.3371	0.0001
cg01285783	*C6orf195*	0.2961	0.0003	0.3076	0.0000
cg15516759	*APOL3*	0.2687	0.0000	0.2741	0.0000

cg18405140	*STK11*	0.2534	0.0255	0.2521	0.0000
cg05928849	*ASAM*	0.2567	0.0077	0.2697	0.0000
cg17256465	*FNDC3B*	0.2681	0.0458	0.2786	0.0181
cg02535635	*CLTC*	0.2904	0.0000	0.3111	0.0000
cg22481673	*RD3*	0.3324	0.0142	0.2661	0.0004
cg13370086	*TTC22*	0.2985	0.0189	0.3138	0.0044
cg00729708	*LASS3*	0.4374	0.0000	0.4625	0.0000
cg18840580	*ESPL1*	0.2852	0.0022	0.2715	0.0000
cg13715467	*GUCY1A2*	0.3563	0.0069	0.3214	0.0043
cg12511848	*GADD45B*	0.3146	0.0007	0.2522	0.0000
cg14265677	*LINC00944*	0.2537	0.0444	0.2695	0.0128
cg07505631	*CDCP1*	0.5261	0.0028	0.3213	0.0098
cg25017994	*MOG*	-0.3089	0.0371	-0.2742	0.0000
cg22053010	*LINC00353*	0.3662	0.0428	0.3381	0.0395
cg08421632	*ANLN*	0.3779	0.0030	0.3735	0.0020
cg12515659	*FAM134B*	0.3377	0.0216	0.3946	0.0075
cg00571519	*SRPK2*	0.3691	0.0198	0.3841	0.0030
cg05834845	*MUC4*	0.3019	0.0000	0.2574	0.0009
cg23178186	*TOLLIP*	0.2674	0.0048	0.2669	0.0000
cg00853216	*SOX6*	0.3536	0.0437	0.3507	0.0172
cg16805094	*THBS2*	-0.2540	0.0235	-0.2571	0.0000
cg24996718	*CLCA2*	-0.3458	0.0000	-0.3872	0.0001
cg12852430	*SOX6*	0.2682	0.0208	0.2825	0.0074
cg18319872	*ITSN1*	0.2838	0.0001	0.2914	0.0000
cg04034998	*ADAM12*	0.2584	0.0398	0.2910	0.0077

For each common DMPs found, their correspondent gene, methylation Beta difference and adjusted-p value is shown. Hyper or hypomethylation determined by the Beta difference refers to the patients' group. Filters applied: adjusted-p value <0.05 and absolute value of Beta difference >0.25. Abbreviations: CTRL, healthy controls; MAPT, GRN, C9orf72, familial frontotemporal dementia caused by mutation in MAPT, GRN or C9orf72; sFTD-Tau, sporadic frontotemporal dementia with tau deposits; sFTD-TDP43, sporadic frontotemporal dementia with TDP43 deposits.
